# Top-Down, Knowledge-Based Genetic Reduction of Yeast Central Carbon Metabolism

**DOI:** 10.1128/mbio.02970-21

**Published:** 2022-09-21

**Authors:** Eline D. Postma, Lucas G. F. Couwenberg, Roderick N. van Roosmalen, Jordi Geelhoed, Philip A. de Groot, Pascale Daran-Lapujade

**Affiliations:** a Department of Biotechnology, Delft University of Technology, Delft, The Netherlands; IMBB-FORTH

**Keywords:** *Saccharomyces cerevisiae*, central carbon metabolism, minimal genome, genetic redundancy

## Abstract

Saccharomyces cerevisiae, whose evolutionary past includes a whole-genome duplication event, is characterized by a mosaic genome configuration with substantial apparent genetic redundancy. This apparent redundancy raises questions about the evolutionary driving force for genomic fixation of “minor” paralogs and complicates modular and combinatorial metabolic engineering strategies. While isoenzymes might be important in specific environments, they could be dispensable in controlled laboratory or industrial contexts. The present study explores the extent to which the genetic complexity of the central carbon metabolism (CCM) in S. cerevisiae, here defined as the combination of glycolysis, the pentose phosphate pathway, the tricarboxylic acid cycle, and a limited number of related pathways and reactions, can be reduced by elimination of (iso)enzymes without major negative impacts on strain physiology. Cas9-mediated, groupwise deletion of 35 of the 111 genes yielded a “minimal CCM” strain which, despite the elimination of 32% of CCM-related proteins, showed only a minimal change in phenotype on glucose-containing synthetic medium in controlled bioreactor cultures relative to a congenic reference strain. Analysis under a wide range of other growth and stress conditions revealed remarkably few phenotypic changes from the reduction of genetic complexity. Still, a well-documented context-dependent role of *GPD1* in osmotolerance was confirmed. The minimal CCM strain provides a model system for further research into genetic redundancy of yeast genes and a platform for strategies aimed at large-scale, combinatorial remodeling of yeast CCM.

## INTRODUCTION

The fundamental challenge of defining the minimum complement of genes required for life has been addressed by theoretical as well as experimental approaches. Bottom-up and top-down strategies have mainly focused on bacteria with small genomes (1–9). Their larger genome sizes might appear to make eukaryotic microorganisms less relevant for this type of research. However, they do offer attractive models to explore the biological significance of (apparent) genetic redundancy. Different evolutionary advantages have been proposed for the fixation of duplicated genes in genomes, including provision of a molecular landscape for functional (minor or major) innovation (e.g., neo- and subfunctionalization), a functional backup, gene dosage effects, or increased buffering to respond to environmental cues (10, 11). Systematically identifying the physiological significance underlying gene fixation presents a daunting challenge.

With its relatively small genome (12 Mb), tractability, and high genetic accessibility, the yeast Saccharomyces cerevisiae is a valuable model for fundamental research on minimal genetic requirements. S. cerevisiae underwent a whole-genome duplication (WGD) approximately 100 million years ago, as well as smaller-scale duplication (SSD) events. While 90% of the WGD genes were lost during evolution, some duplicates remain (11). As observed in humans, a substantial fraction of the total gene duplicates in S. cerevisiae originates from a WGD (approximately 63% of duplicates in the S. cerevisiae genome and 62% in the human genome), while a smaller fraction originates from SSD events (approximately 37% of duplicates in the S. cerevisiae genome and 38% in the human genome) (12, 13). While systematic, large-scale studies like the construction of the yeast deletion collections (14–17), the synthetic genetic array projects (18–20), or the recent SCRaMbLE-based genome compaction (21), have provided valuable information on the dispensability of (a combination of) genes, the physiological roles of many of these paralogous genes remain poorly defined.

In addition to the fundamental scientific questions raised by genetic redundancy, it also complicates genome engineering of S. cerevisiae. The conversion of substrate into product via native or engineered pathways relies on the microbial host core pathways for the supply of metabolic precursors, energy-rich molecules, and redox equivalents. These biochemical reactions are catalyzed by sets of “metabolic” genes that are characterized by a high genetic redundancy in eukaryotes (11). Not only has the physiological role of many paralogous genes not been fully elucidated, but also the manipulation of specific biochemical reactions is hindered by the presence of multiple paralogous genes with poorly known functions that are scattered over the 12-Mb, mosaic yeast genome and its 16 chromosomes. Additionally, expression of these redundant genes dissipates cellular resources (e.g., carbon, energy) that might be better invested in industrially relevant properties, such as high product yield or cellular robustness to the stressful environment of large-scale fermentation.

To tackle these fundamental and applied challenges, taking glycolysis and ethanolic fermentation as starting points, Solis-Escalante and colleagues pioneered the genetic reduction of central carbon metabolism in S. cerevisiae. The set of 26 genes encoding the (iso)enzymes catalyzing 12 reactions was reduced to 13 genes (22). Remarkably, this 50% genetic reduction did not result in any visible phenotypic effect, although a wide range of growth conditions was tested. These observations argued against gene dosage being a strong driving force in the evolution of Crabtree-positive yeasts (11, 22) and raised questions on the mechanisms involved in the fixation of these gene duplicates in the S. cerevisiae genome. A recent study suggested that the role of the redundant paralogs might be highly context dependent and that some relevant conditions were not tested by Solis-Escalante and coworkers (22) (e.g., the role of pyruvate kinase 2 in the utilization of three-carbon substrates such as dihydroxyacetone [23]). The surprising lack of phenotype of the “minimal glycolysis” yeast strain (called MG) enabled the construction of a genetically simplified version of the glycolytic pathway, which was subsequently relocalized to a single chromosomal locus (24). The resulting yeast strain with a single locus for glycolysis presents a powerful tool to remodel the glycolytic pathway in two single steps into any redesigned (heterologous) version.

**FIG 1:**
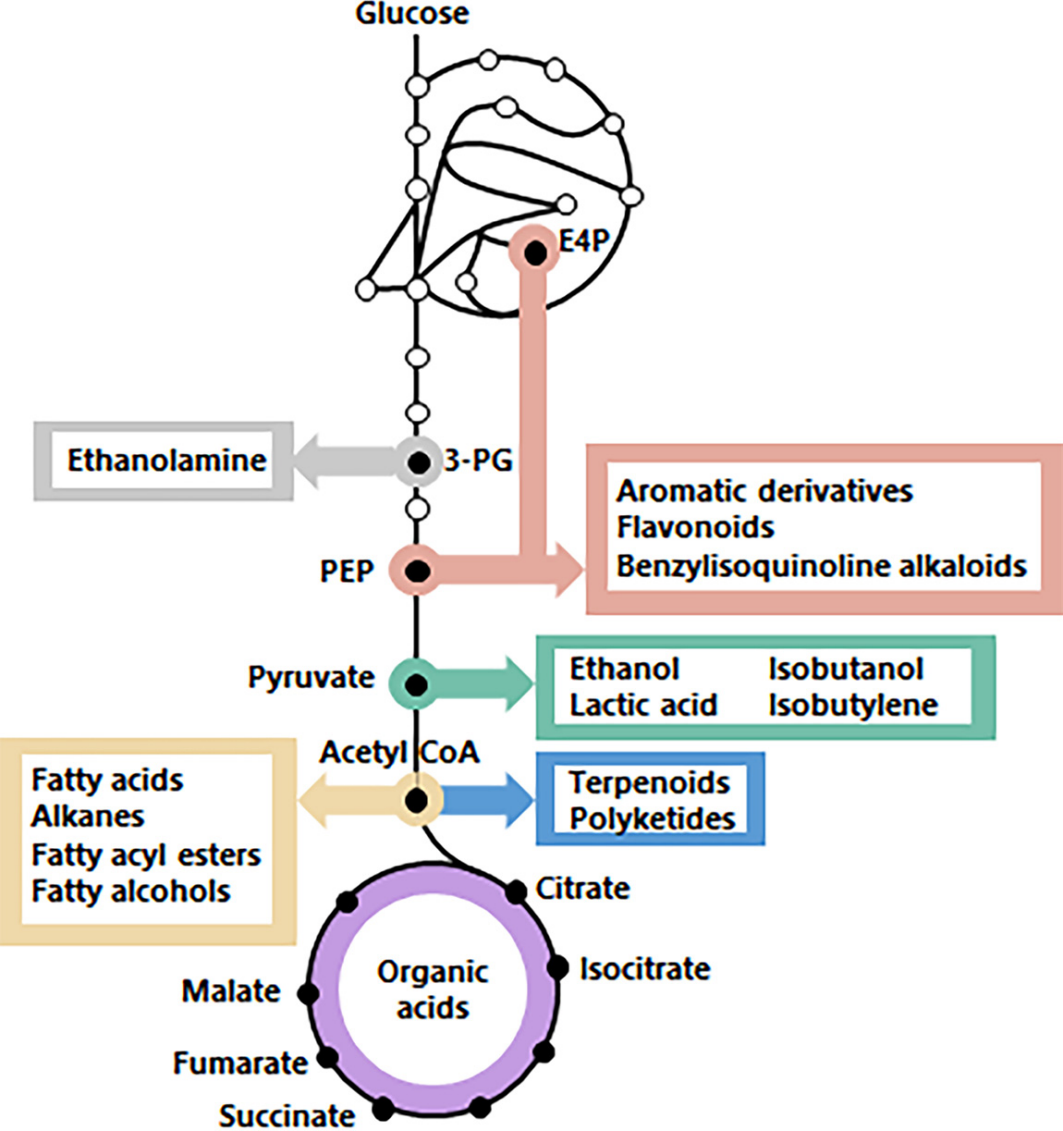
CCM precursors of industrially relevant chemicals. 3-PG, 3-phosphoglycerate; PEP, phosphoenolpyruvate; E4P, erythrose-4-phosphate.

**FIG 2:**
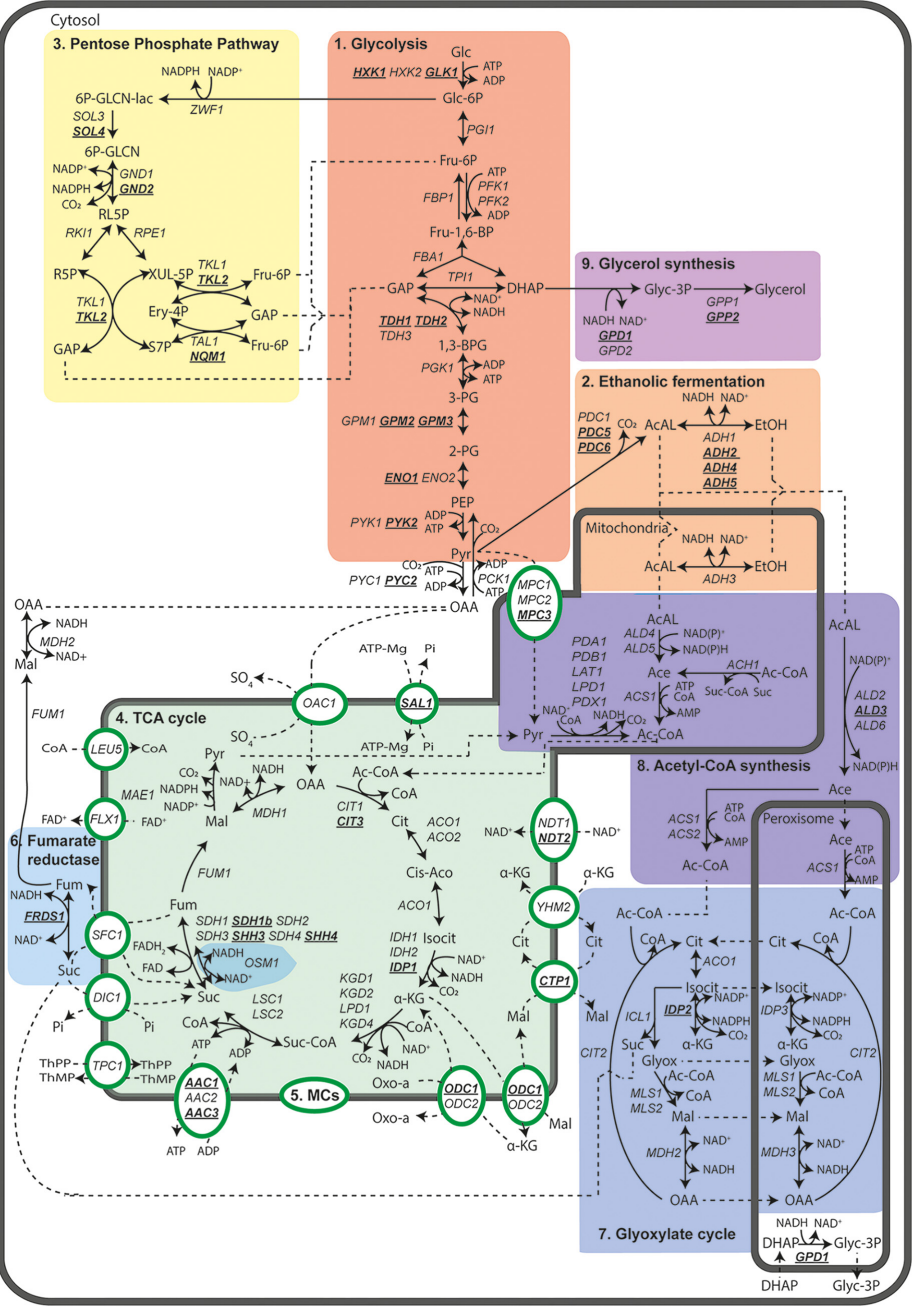
Reactions of the CCM of S. cerevisiae considered for genetic reduction in this study, including subprocesses and pathways in CCM metabolic pathways. Enzyme-catalyzed conversions (black lines) and transport processes (dotted lines) are shown between intermediates and through mitochondrial transporters (circles and ovals), respectively. Directionality and reversibility of reactions were based on information from the Yeast Pathways database (https://pathway.yeastgenome.org/). Enzyme localization was based on literature information. Genes retained in the genetic reduction strategy are shown in black, and genes selected for deletion in the minimal CCM strain are indicated in bold and underlined. Occurrence of pathways in different cellular compartments is shown by gray borders. Simplifications have been made for visualization reasons, for example, H_2_O and inorganic phosphate are not shown. 6P-GLCN-lac, 6-phosphogluconolactone; 6P-GLCN, 6-phosphoglucononate; RL5P, ribulose 5-phosphate; R5P, ribose 5-phosphate; XUL-5P, xylulose 5-phosphate; Fru-6P, fructose-6-phosphate; Ery-4P, erythrose 4-phosphate; S7P, sedoheptulose 7-phosphate; GAP, glyceraldehyde-3-phosphate; Glc, glucose; Glc-6P, glucose-6-phosphate; Fru-1,6-BP, fructose-1,6-bisphosphate; DHAP, dihydroxyacetone phosphate; 1,3-BPG, 1,3-bisphosphoglycerate; 3-PG, 3-phosphoglycerate; 2-PG, 2-phosphoglycerate; PEP, phosphoenolpyruvate; Pyr, pyruvate; AcAl, acetaldehyde; EtOH, ethanol; OAA, oxaloacetate; Cit, citrate; Cis-Aco, *cis*-aconitate; Isocit, isocitrate; α-KG, α-ketoglutarate; Suc-CoA, succinyl-CoA; Suc, succinate; Fum, fumarate; Mal, malate; Glyox, glyoxylate; Ace, acetate; Ac-CoA, acetyl-CoA; Glyc-3P, glycerol-3-phosphate.

Glycolysis is an important but small part of central carbon metabolism (CCM), a set of reactions required for the conversion of carbon feedstocks into any industrially relevant product (Fig. 1). For cells, CCM is primarily the set of reactions that convert carbon sources into the 12 building blocks required for the synthesis of cellular components (25). CCM encompasses ~111 genes, with 66% duplicates (Fig. 2). Reducing its genetic complexity would be the first step in an attempt to construct a modular, designer yeast genome, with a single-locus CCM, as previously achieved for glycolysis and fermentation. Modular, specialized synthetic chromosomes could be ideal platforms for the centralization of the CCM genes (26).

The main goal of this study was to explore the extent to which the number of genes encoding CCM enzymes in S. cerevisiae can be reduced without substantially affecting fitness under a set of chosen growth conditions. To this end, redundancies were first predicted based on data in the literature on gene expression, enzyme activities, and phenotypes of (single) deletion mutants. Subsequently, phenotypes of mutants with mutations in sets of genes encoding CCM enzymes were tested under a wider range of growth conditions. In this first attempt of genetic reduction of yeast CCM at this scale, special attention was given to possible synergistic effects of mutations that were previously studied in separate strains.

## RESULTS

### Genetic reduction strategy.

In this study, the CCM of S. cerevisiae was defined as the set of biochemical reactions encompassed by glycolysis, ethanolic fermentation, pentose-phosphate pathway (PPP), acetyl-coenzyme A (CoA) synthesis, tricarboxylic acid cycle (TCA), anaplerosis, gluconeogenesis, glyoxylate cycle, and glycerol metabolism. As CCM reactions occur in multiple compartments, mitochondrial transporters were also considered (Fig. 2). Transport through the peroxisomal membrane was not considered, as this phenomenon is poorly studied (27). For the construction of a minimal CCM strain, decisions to remove or retain genes were based on (i) transcript levels from an expression compendium encompassing 170 different cultivation conditions (28), (ii) enzyme activities in cell extracts of mutant strains when data were available, and (iii) reported phenotypes of null mutants. Genes encoding proteins with reported secondary (“moonlighting”) functions or proteins known to cause auxotrophy upon deletion were retained (29).

Genes were classified as functionally redundant when at least 75% of the specific growth rate of the congenic reference strains CEN.PK113-7D (Ura^+^) or IMX581 (Ura^−^) was retained during aerobic batch cultivation on synthetic medium supplied with either glucose or ethanol. Ethanol-grown cultures were included because, in contrast to glucose, ethanol can only be dissimilated by respiration and because its metabolism involves different sets of CCM enzymes and transporters. In addition, testing for growth on ethanol ensured that the intensive engineering undergone by the strains, including removal of several mitochondrial proteins, did not cause respiratory deficiency.

**FIG 3:**
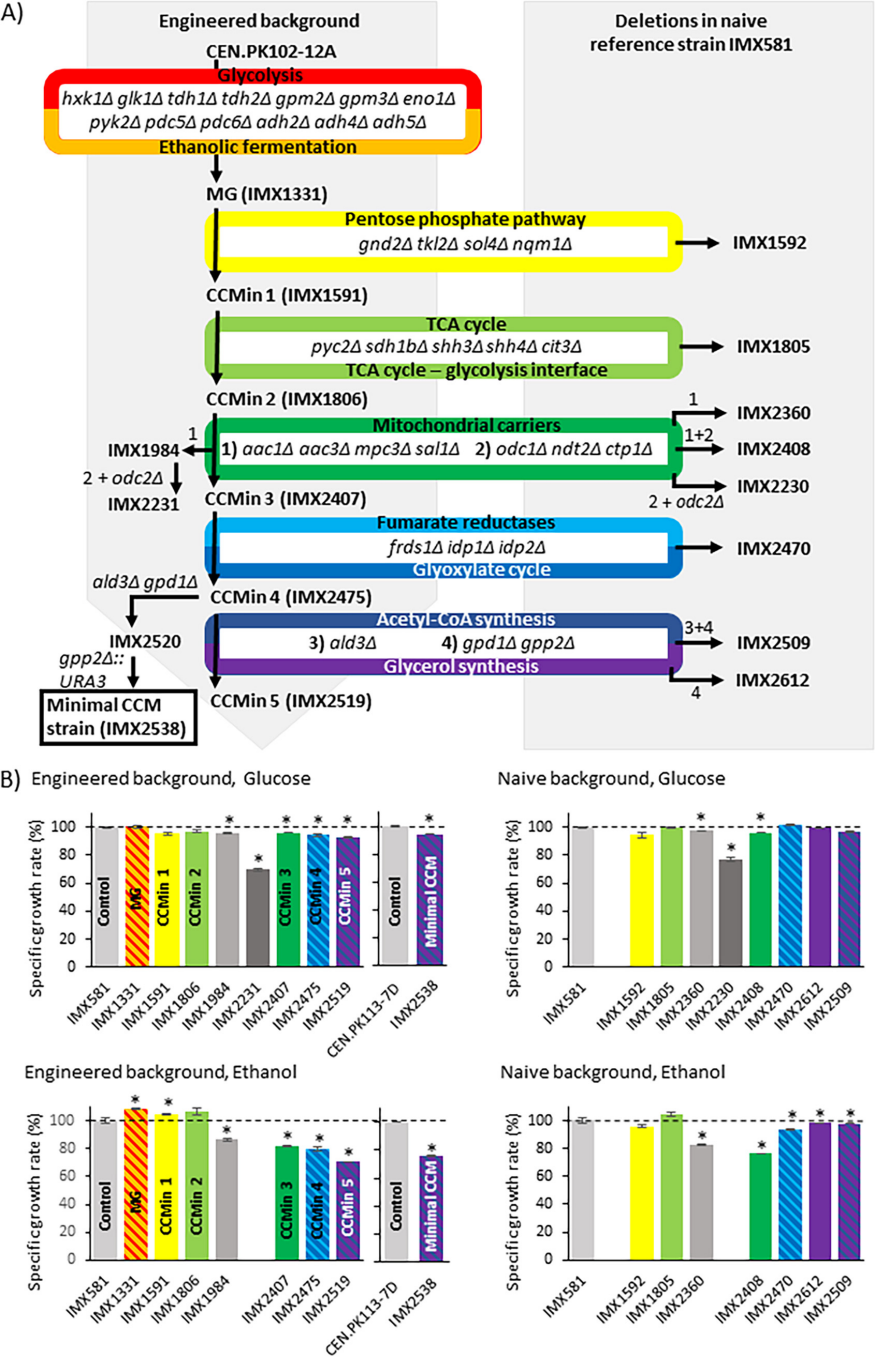
Deletion strategy and specific growth rates of resulting strains. (A) Workflow for construction of relevant S. cerevisiae strains. (B) The strains’ respective specific growth rates, measured as shake flask growth rate on synthetic medium with glucose (SMD) or ethanol (SME) as carbon source, supplemented with uracil. Specific growth rates represent averages and standard deviations of measurements on independent duplicate cultures for each strain and are expressed as a percentage of specific growth rate of the naive uracil-auxotrophic reference strain S. cerevisiae IMX581 or the naive uracil-prototrophic reference strain S. cerevisiae CEN.PK113-7D. Significant differences in specific growth rates relative to the control strain are indicated with an asterisk (*P* < 0.05; two-tailed paired homoscedastic *t* test).

The previously constructed MG strain, in which 13 out of the 26 existing paralogs of genes encoding glycolytic enzymes and fermentation enzymes were deleted without the detection of major phenotypes, was used as a starting point of the present CCM reduction endeavor. To identify any synergistic effects between the newly introduced deletions and the 13 deletions already present in the MG strain, a congenic naive reference strain (IMX581) with a full complement of glycolytic and fermentation genes was also used in parallel to MG for serial deletions. To accelerate the deletion workflow, genes involved in individual pathways or processes were deleted in sets of two to four (Fig. 3). When a substantial loss of fitness was observed, the contribution of individual deletions was dissected by constructing additional strains with various combinations and numbers of deletions.

Deletion of 35 CCM genes had minimal impact on specific growth rates on chemically defined glucose medium.

### Pentose-phosphate pathway.

The pentose-phosphate pathway reduces cellular NADP^+^, generates ribose-5-phosphate and erythrose-4-phosphate for nucleic acid and amino acid synthesis and, in strains engineered for pentose fermentation, acts as a dissimilatory pathway (30).

Four of the seven reactions in the PPP are catalyzed by pairs of isoenzymes encoded by WGD paralogs, for which sequence similarities ranged from 47% (*SOL3* and *SOL4*) to 87% (*GND1* and *GND2*) (see Table S1 in the supplemental material). Based on transcript levels across a wide range of cultivation conditions (28), *SOL4*, *GND2*, *TKL2*, and *NQM1* were considered minor paralogs. Moreover, deletion of *TKL2* and *NQM1* was previously reported not to affect growth on glucose synthetic medium (31–33). While similar *in vitro* enzyme activities were reported for Sol3 and Sol4 in cell extracts (34), *SOL4* was deleted based on its consistently lower transcript level (28). Simultaneous deletion of *GND2*, *TKL2*, *SOL4*, and *NQM1* in the naive reference strain or in the MG strain, while retaining *SOL3*, *GND1*, *TKL1*, and *TAL1*, did not significantly affect growth rate on either synthetic medium with 2% (wt/vol) glucose (SMD) or 2% (vol/vol) ethanol (SME) as carbon source (strains IMX1592 [ppp^min^] and IMX1591 [CCMin1, glyc^min^ fer^min^ ppp^min^]) (ppp^min^, minimized pentose phosphate pathway; glyc^min^, minimized glycolysis; fer^min^, minimized ethanolic fermentation) (Fig. 3). Previously reported extended lag phase and slower growth on ethanol of *sol4* null mutants (35) were not observed. This difference may have been related to the use of different S. cerevisiae strain backgrounds.

10.1128/mBio.02970-21.4 TABLE S1 Genetic characteristics of the paralogs considered for deletion. Download Table S1, PDF file, 0.1 MB. Copyright © 2022 Postma et al. 2022 Postma et al. https://creativecommons.org/licenses/by/4.0/ This content is distributed under the terms of the Creative Commons Attribution 4.0 International license.

### Tricarboxylic acid cycle, anaplerotic reactions, and gluconeogenesis.

In addition to its dissimilatory role in oxidizing acetyl-CoA units to CO_2_, the TCA cycle supplies precursors, NADH, FADH_2_, and ATP (36). During growth on fermentable sugars, the TCA cycle is a mitochondrial pathway, with acetyl-CoA resulting from oxidative decarboxylation of pyruvate by the pyruvate dehydrogenase complex. To replenish use of TCA cycle intermediates for biosynthesis, the cycle’s acceptor molecule, oxaloacetate, can be imported from the cytosol, where it is produced by carboxylation of pyruvate. The nine biochemical reactions of the TCA cycle involve 22 mitochondrial enzymes, which show little genetic redundancy. Two reactions are catalyzed by single enzymes, Mdh1 and Fum1, while three steps are catalyzed by complexes of two to five proteins. Deletion of genes encoding individual subunits of the α-ketoglutarate and succinyl-CoA synthetase complexes renders the complexes dysfunctional (37–42). In contrast, the succinate dehydrogenase complex, in which four functions are performed by seven proteins, does show some redundancy. *SDH1*, *SDH3*, and *SDH4* proteins have the genes *SDH1b*, *SHH3*, and *SHH4*, respectively, as homologs originating from the WGD, while *SDH2* is a unique gene (43–48). Deletion of *SDH1b*, *SHH3*, and *SHH4* has a minor or no effect on complex integrity and yeast physiology, and these genes are considered functionally redundant (45, 48, 49). Citrate synthase (Cit1 and Cit3) and isocitrate dehydrogenase (Idh1, Idh2, and Idp1) have functionally redundant mitochondrial enzymes. Based on expression data and lack of a phenotypic difference during fermentative and respiratory growth, Cit3 and the NADP^+^-dependent Idp1 are considered redundant (28, 50–52). Single deletion of *ACO1* or *ACO2*, which encode aconitase isoenzymes, causes amino acid auxotrophies (53, 54). Idh1 and Idh2 are part of a complex and are both required for isocitrate dehydrogenase activity (37, 38). Based on this information, only 5 of the 22 TCA cycle mitochondrial proteins were considered functionally redundant and, therefore, selected as candidates for elimination: Cit3, Idp1, Sdh1b, Shh3, and Shh4. *IDP1* was targeted in a later deletion round, along with extramitochondrial paralogs of the TCA cycle that are part of the glyoxylate cycle.

There are several enzymes that form an interface between the TCA cycle and glycolysis. The WGD paralog pair *PYC1* and *PYC2* encodes isoenzymes of the anaplerotic enzyme pyruvate carboxylase. Transcript levels of these two highly similar genes (92%) (see Table S1) are condition dependent, and despite some conflicting reports on the physiological impact of *PYC1* and *PYC2* deletion (28, 55–57), one study showed that only deletion of *PYC1* leads to aspartate auxotrophy (58). *PYC2* was therefore deleted. Deletion of *MAE1*, which encodes a mitochondrial malic enzyme catalyzing the oxidative decarboxylation of malate to pyruvate, does not show a clear phenotype. However, double deletion of *MAE1* and *PYK2* reduces the specific growth rate on ethanol by 62% (58). As *PYK2* was deleted in the MG strain, *MAE1* was retained. The gluconeogenic enzymes phosphoenolpyruvate carboxykinase (Pck1) and fructose-1,6-bisphosphatase (Fbp1) are essential for bypassing the irreversible pyruvate kinase and phosphofructokinase reactions, respectively, during growth on nonfermentable carbon sources (59, 60).

*CIT3*, *SDH1b*, *SHH3*, *SHH4*, and *PYC2* were deleted in two consecutive transformation rounds in the naive reference strain and the CCMin1 strain (glyc^min^ fer^min^ ppp^min^), resulting in IMX1805 (tca^min^) and IMX1806 (CCMin2, glyc^min^ fer^min^ ppp^min^ tca^min^), respectively. Both strains grew as well as their parental strains in chemically defined medium supplemented with glucose or ethanol (Fig. 3).

### Mitochondrial carriers.

The 35 nuclearly encoded mitochondrial carriers (MCs) mediate transport of numerous metabolites, nucleotides, cofactors, and inorganic anions between the mitochondrial matrix and cytosol (61). Based on extensive functional analysis studies (62, 63), 19 MCs involved in transport of pyruvate, TCA cycle intermediates CoA, ADP, ATP, P_i_, NAD^+^, FAD^+^, and thiamine pyrophosphate (a cofactor of pyruvate dehydrogenase and α-ketoglutarate dehydrogenase) were considered part of CCM (Fig. 2; see also Table S2). Potential genetic redundancy was identified for 10 of these MCs, with protein sequence similarity varying between 51 and 87% (see Table S1). In addition to genetic redundancy, functional redundancy has to be considered, since several genetically distinct transporters can transport the same solutes, as exemplified by the antiport of ADP and ATP across the mitochondrial membrane by three Aac isoforms as well as by Sal1. Aac2 and Aac3 originate from WGD, while Aac1 does not. Sal1 shares no homology with the Aac carriers and harbors an additional Ca^2+^-binding domain (64, 65). Several studies indicate Aac2 as a major paralog whose presence suffices to sustain adenine nucleotide transport during respiratory growth (28, 64–69). *AAC1*, *AAC3*, and *SAL1* were therefore all candidates for deletion. NAD^+^, synthesized in the cytosol and required for the NAD^+^-dependent mitochondrial dehydrogenases in CCM, is imported by two MCs encoded by *NDT1* and *NDT2*, paralogs with 51% similarity at the protein level (see Table S1). *NDT1* and *NDT2* are individually dispensable for growth on glucose or ethanol, but deletion of both precludes growth on nonfermentable carbon sources (70). Therefore, only one of the paralogs, *NDT2*, was chosen for deletion. Since import of FAD^+^, CoA, and thiamine pyrophosphate is crucial for mitochondrial activity, the corresponding unique genes (*FLX1*, *LEU5*, and *TPC1*) were retained (71–74).

10.1128/mBio.02970-21.5 TABLE S2 Mitochondrial carrier proteins. Download Table S2, PDF file, 0.1 MB. Copyright © 2022 Postma et al. 2022 Postma et al. https://creativecommons.org/licenses/by/4.0/ This content is distributed under the terms of the Creative Commons Attribution 4.0 International license.

Pyruvate is located at the interface of glycolysis and the TCA cycle and, in addition, mitochondrial pyruvate is required for synthesis of branched-chain amino acids (BCAA). Pyruvate import into mitochondria is mediated by three isoforms: Mpc1, Mpc2, and Mpc3. Mpc1 is constitutively expressed and forms complexes with either of the highly homologous Mpc2 or Mpc3 (75). *MPC2* is expressed during fermentative growth, while *MPC3* is expressed during respiratory growth. Deletion of *MPC2* leads to a severe growth defect, even in glucose-containing medium supplied with BCAA, while *MPC3* deletion leads to a modest (20%) decrease of specific growth rates on nonfermentable carbon sources (76, 77). Based on these literature data, it was decided to delete *MPC3*.

Sfc1 and Dic1 employ different mechanisms to import succinate into mitochondria and, since both are essential for growth on ethanol (78–80), neither was eliminated. Oxaloacetate is mainly transported by Oac1, whose removal only has a minor impact on specific growth rate on glucose medium, which is linked to its secondary function as an exporter of α-isopropylmalate for leucine biosynthesis (81, 82). Since we observed a 26% reduction of the specific growth rate on glucose upon deletion of *OAC1* in the CEN.PK genetic background used in this study (see Fig. S1), it was retained in strain construction.

10.1128/mBio.02970-21.1 FIG S1 Growth rate of the *OAC1* deletion mutant. Maximum specific growth rate of S. cerevisiae CEN.PK113-7D (naive reference strain) and the *OAC1* deletion mutant, S. cerevisiae IMK588, tested in shake flasks on selective SMD medium. Growth rates represent averages and standard deviations of two biological duplicates. *, *P* < 0.05; IMK588 had a 26% slower growth rate with respect to CEN.PK113-7D (two-tailed paired homoscedastic t test). Download FIG S1, PDF file, 0.2 MB. Copyright © 2022 Postma et al. 2022 Postma et al. https://creativecommons.org/licenses/by/4.0/ This content is distributed under the terms of the Creative Commons Attribution 4.0 International license.

Four additional and partially functionally redundant MCs with different transport mechanisms and affinities mediate organic acid transport. Ctp1 is a citrate-malate antiporter, the paralogous carriers Odc1 and Odc2 are α-ketoglutarate and oxodicarboxylate antiporters, and Yhm2 exchanges α-ketoglutarate and citrate, thereby enabling NADPH shuttling between cytosol and mitochondria (involving isocitrate dehydrogenase and aconitase) (83–89). Deletion of *CTP1* or double deletion of *ODC1* and *ODC2* does not affect growth, while triple deletion of *YHM2*, *ODC1*, and *ODC2* does (84, 85). Based on these literature data, *CTP1*, *ODC1*, and *ODC2* were selected for deletion, with the realization that their combined deletion might affect di- and tricarboxylic acid trafficking.

In total, 8 MCs were targeted for elimination. First, *AAC1*, *AAC3*, *SAL1*, and *MPC3* were simultaneously deleted, followed by simultaneous deletion of *NDT2*, *CTP1*, *ODC1*, and *ODC2*. Deletion of *AAC1*, *AAC3*, *SAL1*, and *MPC3* in the naive reference strain, resulting in strain IMX2360, only marginally affected the specific growth rate on SMD (3 to 5% decrease) but had a stronger impact on growth on SME (14 to 18% slower growth) (Fig. 3). These results are in agreement with the reported roles of these MCs in respiratory growth. When introduced in CCMin2 (glyc^min^ fer^min^ ppp^min^ tca^min^), resulting in strain IMX1984, the same set of deletions did not affect the specific growth rate on either SMD or SME.

Combined deletion of *NDT2*, *CTP1*, *ODC1*, and *ODC2* reduced the specific growth rate on SMD by 23% in the naive reference strain (resulting in strain IMX2230) and by 30% in IMX1984 (glyc^min^ fer^min^ ppp^min^ tca^min^ *aac1Δ aac3Δ sal1Δ mpc3Δ*; resulting in strain IMX2231) (Fig. 3). When instead only *NDT2*, *CTP1*, and *ODC1* were deleted in the naive reference strain (resulting in strain IMX2404) or in engineered background strain IMX1984 (resulting in strain IMX2407, called CCMin3: glyc^min^ fer^min^ ppp^min^ tca^min^ mc^min^; mc^min^ is an abbreviation for minimized mitochondrial carriers), the specific growth rate on SMD was not affected and only a small (3 to 7%) reduction of growth rate was observed on SME (Fig. 3; see also Fig. S2). CCMin3 (glyc^min^ fer^min^ ppp^min^ tca^min^ mc^min^) retained 96% of the specific growth rate of the reference strain on SMD and 82% of its specific growth rate on SME.

10.1128/mBio.02970-21.2 FIG S2 Growth rates of deletion strains. Maximum specific growth rates of all S. cerevisiae strains tested in shake flasks on selective SMD (A and C) or SME (B and D) supplemented with uracil. Represented are deletion strains in the naive background (A and B) and in the engineered background (C and D). Color coding matches that used in Fig. 2 in the main article. Growth rates represent averages and standard deviations of two biological duplicates. Growth rates of the deletion strains are expressed as percentages of the control strain IMX581 growth rate. Significant changes in growth rate with respect to the control strain are indicated (*, *P* < 0.05, two-tailed paired homoscedastic *t* test). Download FIG S2, PDF file, 0.3 MB. Copyright © 2022 Postma et al. 2022 Postma et al. https://creativecommons.org/licenses/by/4.0/ This content is distributed under the terms of the Creative Commons Attribution 4.0 International license.

### Fumarate reductases, acetyl-CoA synthesis, and glyoxylate cycle.

Cytosolic (Frds1) and mitochondrial (Osm1) fumarate reductases reoxidize FADH_2_, which has been proposed to be important for protein folding under anaerobic conditions (90–93). Double deletion of *FRDS1* and *OSM1* has no phenotypic effect on complex glucose medium under aerobic conditions (94). However, Osm1 has a moonlighting function outside CCM, as it contains two translation sites, leading to the targeting to the endoplasmic reticulum of an Osm1 variant. Therefore, only *FRDS1* was considered for deletion in the design of a minimal CCM strain.

The glyoxylate cycle, which is essential for providing biosynthetic precursors with more than 2 carbon atoms during growth on fatty acids and two-carbon compounds, encompasses reactions in the peroxisome and cytosol (95, 96) and uses acetyl-CoA as substrate, which is made by the acetyl-CoA synthesis pathway. Ethanol is converted into to acetyl-CoA via alcohol dehydrogenase (already reduced in the MG strain), acetaldehyde dehydrogenases, and acetyl-CoA synthetases. Five acetaldehyde dehydrogenase isoenzymes, Ald2 to Ald6, oxidize acetaldehyde to acetate with either NADP^+^ or NAD^+^ as cofactor. The mitochondrial isoenzymes Ald4 and Ald5, required for growth on ethanol (97, 98) and for maintenance of a functional respiratory chain (98), were both retained. Ald6 is the major cytosolic isoenzyme whose elimination strongly affects growth on fermentable and nonfermentable carbon sources (99). The other two cytosolic acetaldehyde dehydrogenases, Ald2 and Ald3, are involved in conversion of 3-aminopropanal to β-alanine for pantothenic acid biosynthesis (100, 101). As single deletion of *ALD2* or *ALD3* does not affect growth on ethanol or glucose and *ALD2* is the major paralog in pantothenic acid production (100, 101), *ALD3* was considered for deletion.

Acetate is then converted to acetyl-CoA via Acs1, whose localization is under debate. Acs1 has been reported to occur in the cytosol, nucleus, and peroxisomes, depending on growth conditions (102, 103). Acs1 and its isoenzyme Acs2 are essential for growth on nonfermentable and fermentable carbon sources, respectively (103). The mitochondrial acetyl-CoA hydrolase Ach1 is also able to convert acetate into acetyl-CoA but uses succinyl-CoA as CoA donor. Deletion of *ACH1* leads to reduced chronological life span, severe mitochondrial damage, and accumulation of reactive oxygen species (104, 105). *ACS1*, *ACS2*, and *ACH1* were therefore retained.

The glyoxylate cycle is initiated by Cit2, an extramitochondrial isoenzyme of the mitochondrial Cit1 and Cit3 citrate synthases, whose localization is under debate and has been reported in the cytosol and peroxisome (103, 106). Citrate is then converted into isocitrate in the cytosol by the dually localized enzyme Aco1 in cytosol and mitochondria (107). Via a series of cytosolic and peroxisomal reactions (some localizations under debate), including the isocitrate lyase Icl1 (cytosol) (108, 109), the malate synthase (Mls1 or Mls2, cytosol and peroxisome) (103, 110, 111), and malate dehydrogenase (Mdh2 in cytosol and Mdh3 occurs in the peroxisome) (111), the net synthesis of TCA cycle intermediates is enabled from acetyl-CoA.

Possible redundancies of glyoxylate enzymes also involved in the TCA cycle were discussed above, with only Cit3 selected for elimination in a minimal CCM strain. Since the glyoxylate cycle enzymes Cit2, Mls1, Icl1, and Mdh2 (cytosolic isoenzyme of the mitochondrial Mdh1) are either essential for growth on C_2_ compounds or their elimination leads to strong reductions in growth rate, they were retained in the minimal CCM design (95, 103, 112–116). The proteins Icl2, Mls2, and Mdh3 are homologous to Icl1, Mls1, and Mdh1 or Mdh2, respectively, but have (additional) functions outside the CCM (113, 117, 118) and were therefore also retained.

The peroxisomes harbor the NADP^+^-dependent isocitrate dehydrogenase Idp3. Deletion of its mitochondrial homologs Idp1 and Idp2 does not affect growth on ethanol or glucose (51, 52). Idp1 and Idp2 were therefore the only genes considered for elimination in the minimal CCM design.

Triple deletion of *FRDS1*, *IDP1*, and *IDP2* in the naive reference strain (resulting in strain IMX2470, fum^min^ glyox^min^) (fum^min^, minimized fumarate reductases; glyox^min^, minimized glyoxylate cycle) did not affect the specific growth rate on SMD and caused a 7% lower growth rate on SME (Fig. 3; see also Fig. S2). Deletion in CCMin3 (IMX2407, glyc^min^ fer^min^ ppp^min^ tca^min^ mc^min^) did not affect the specific growth rate on either SMD or SME (Fig. 3). The resulting strain, CCMin4 (IMX2475, glyc^min^ fer^min^ ppp^min^ tca^min^ mc^min^ fum^min^ glyox^min^), retained 94% and 79% of the specific growth rate of the naive reference strain IMX581 on SMD and SME, respectively. For reasons of experimental efficiency, *ALD3* was removed in the final deletion round (see below).

### Glycerol synthesis.

Glycerol production is essential for redox balancing in anaerobic S. cerevisiae cultures (119). In addition, glycerol plays a key role in osmotolerance and maintenance of cellular volume and turgor pressure during growth under hypertonic conditions (120, 121). The conversion of dihydroxyacetone phosphate to glycerol-3-phosphate is catalyzed by the isoenzymes Gpd1 and Gpd2. *gpd1* deletion mutants are osmosensitive but show no growth defects in the absence of stress (122–124). In contrast, *gpd2* null mutants show a mild reduction of aerobic growth rates and strongly decreased growth rates under anaerobic conditions (125, 126). Glycerol-3-phosphate is converted into glycerol by the redundant Gpp1 and Gpp2 isoenzymes. Single deletion of either enzyme affected neither osmotolerance nor growth on glucose or ethanol, while *gpp1* mutants have been reported to show extended lag phases in anaerobic cultures (127, 128). Therefore, *GPD1* and *GPP2* were chosen for deletion.

Triple deletion of *ALD3*, *GPD1*, and *GPP2* did not significantly affect the specific growth rate on SMD, while a small growth rate reduction was observed on SME in both the naive reference strain and the engineered background (IMX2509 [ace^min^ glycerol^min^] and IMX2519 [CCMin5, glyc^min^ fer^min^ ppp^min^ tca^min^ mc^min^ fum^min^ glyox^min^ ace^min^ glycerol^min^], respectively) (ace^min^, minimized acetyl-CoA synthesis; glycerol^min^, minimized glycerol synthesis) (Fig. 3). The lower specific growth rate on SME could be attributed to the double deletion of *GPD1* and *GPP2* (strain IMX2612) (Fig. 3; see also Fig. S2).

The auxotrophic 35-deletion strain IMX2519 (glyc^min^ fer^min^ ppp^min^ tca^min^ mc^min^ fum^min^ glyox^min^ ace^min^ glycerol^min^) grew at 93% and 71% of the specific growth rate of the control strain IMX581 on uracil-supplemented SMD and SME, respectively. Integration of a *URA3* cassette yielded the uracil-prototrophic 35-deletion strain IMX2538, which was labeled the minimal CCM strain. This prototrophic strain grew at 94% of the rate of the prototrophic control strain with a full set of CCM genes, CEN.PK113-7D, on SMD and at 76% of the control on SME (Fig. 3). These values were within the 25% boundary that were initially set, and the physiology of the minimal CCM strain was further explored.

### An S. cerevisiae strain with minimalized CCM shows only mild growth defects on synthetic media.

The genome sequence of the minimal CCM strain was analyzed by short-read and long-read techniques. Long-read sequencing revealed that 9 transformation rounds and deletion of 22 genes from the MG strain had not led to chromosomal rearrangements or deletions. Previously reported duplicated regions on chromosomes 3 and 5 of the MG strain, based on karyotyping and short read sequencing (22), were also observed in this study with long-read sequencing. Sequence analysis confirmed that all 22 targeted CCM genes were correctly deleted from the MG strain. The genome of the minimal CCM strain showed 45 single-nucleotide polymorphisms (SNPs) relative to the MG strain, of which 8 were located in genes and only 4 led to an amino acid change (Table 1), none of which affected proteins involved in CCM.

**TABLE 1:** Single-nucleotide mutations identified in coding regions of the prototrophic minimal CCM strain IMX2538^*a*^

Systematic name	Gene name	Type^*b*^	Amino acid change
YBR114W	*RAD16*	NS	Ile-202-Thr
YDL035C	*GPR1*	S	Asn-523-Asn
YDR098C	*GRX3*	NS	Glu-239-Asp
YFL062W	*COS4*	S	Cys-151-Cys
YLR002C	*NOC3*	NS	Asp-526-Glu
YML058W	*SML1*	NS	Gly-52-Ser
YMR154C	*RIM13*	S	Lys-265-Lys
YNL273W	*TOF1*	S	Asn-117-Asn

a Single-nucleotide changes in S. cerevisiae IMX2538 (prototrophic minimal CCM) relative to the genome sequence of S. cerevisiae IMX372 (prototrophic minimal glycolysis (MG) (22) are shown.

b S, synonymous; NS, nonsynonymous.

The physiology of the minimal CCM strain was compared to that of the congenic reference strain CEN.PK113-7D, which has a full complement of CCM genes, in pH-controlled aerobic bioreactor cultures on SMD. Consistent with the analyses in shake flasks, the specific growth rate of the minimal CCM strain in these cultures was 8% lower than the level of the reference strain CEN.PK113-7D (Table 2). During the glucose consumption phase, biomass-specific glucose and oxygen consumption rates of the two strains, as well as their ethanol and CO_2_ production rates and their biomass and ethanol yields on glucose, were also similar. The minimal CCM strain did exhibit a higher acetate production rate and yield (63% and 71% higher, respectively) than the reference strain, a difference already observed for the MG strain (22). Similarly, a lower glycerol production rate and glycerol yield on glucose (27% and 23% lower, respectively) was in line with data reported for a *gpd1* deletion mutant (122). After the diauxic shift, growth of the minimal CCM strain on ethanol, glycerol, and organic acids produced during the glucose phase proceeded at a 17% lower rate than observed for the reference strain (Table 2). As a macroscopic characterization based on extracellular products might mask subtle differences of intracellular fluxes, intracellular concentrations of CCM intermediates were measured during the mid-exponential growth phase on glucose. Despite higher T6P and NAD^+^ concentrations than previously reported (129, 130), the concentrations of these metabolites hardly differed between the minimal CCM and the control strains (Table 3). These results indicated that a 32% reduction of the complement of genes encoding CCM enzymes of S. cerevisiae had only a small impact on its physiology under standard laboratory conditions.

**TABLE 2:** Physiological characterization of a 35-deletion, minimal CCM prototrophic S. cerevisiae strain in aerobic bioreactor batch cultures^*a*^

Growth phase and factor	CEN.PK113-7D (naive reference)	IMX372 (minimal glycolysis)	IMX2538 (minimal CCM)
Glucose phase (μ_max_, h^−1^)	0.37 ± 0.00	0.38 ± 0.01*	0.34 ± 0.00*
q_s_ (mmol g_DW_^−1^ h^−1^)	−16.2 ± 0.2	−15.7 ± 0.7	−15.4 ± 0.5
qEthanol (mmol g_DW_^−1^ h^−1^)	23.5 ± 1.5	23.1 ± 1.1	23.2 ± 2.1
qGlycerol (mmol g_DW_^−1^ h^−1^)	1.52 ± 0.05	1.40 ± 0.02	1.11 ± 0.05*
qAcetate (mmol g_DW_^−1^ h^−1^)	0.44 ± 0.03	0.82 ± 0.04*	0.71 ± 0.02*
qCO_2_ (mmol g_DW_^−1^ h^−1^)	23.4 ± 0.2		22.6 ± 0.5
qO_2_ (mmol g_DW_^−1^ h^−1^)	−6.8 ± 0.4		−7.0 ± 0.2
Y_biomass/glucose_ (g_DW_ g_glucose_^−1^)	0.13 ± 0.00	0.13 ± 0.00	0.12 ± 0.00
Y_ethanol/glucose_ (mol mol^−1^)	1.45 ± 0.09	1.48 ± 0.01	1.51 ± 0.09
Y_glycerol/glucose_ (mol mol^−1^)	0.09 ± 0.00	0.09 ± 0.01	0.07 ± 0.00*
Y_acetate/glucose_ (mol mol^−1^)	0.03 ± 0.00	0.05 ± 0.00	0.05 ± 0.00*
Postdiauxic phase			
μ_max_ (h^−1^)	0.10 ± 0.00	0.12 ± 0.00*	0.08 ± 0.01*
qEthanol (mmol g_DW_^−1^ h^−1^)	−3.10 ± 0.19	−3.93 ± 0.04*	−3.07 ± 0.31

a S. cerevisiae strains were grown at pH 5.0 and at 30°C in aerobic bioreactors on synthetic medium with glucose as sole carbon source. Data are presented as averages and standard deviations of 3 biological replicates for S. cerevisiae strains CEN.PK113-7D (naive reference) and IMX2538 (minimal CCM). DW, dry weight. Data for S. cerevisiae IMX372 (minimal glycolysis) were recalculated from the raw data of Solis-Escalante et al. (22) and were obtained with two biological replicates. Statistical significance with respect to CEN.PK113-7D is indicated in boldface with an asterisk (*P* < 0.05, two-tailed *t* test, equal variances).

**TABLE 3:** Intracellular metabolite profiles of a 35-deletion, minimal CCM prototrophic S. cerevisiae strain in aerobic bioreactor batch cultures^*a*^

Pathway and metabolite	Amount of metabolite (μmol/g biomass [dry wt]^−1^)		Fold difference
	CEN.PK113-7D (naive reference)	IMX2538 (minimal CCM)	
Glycolysis			
Glucose 6-phosphate	4.13 ± 0.49	5.01 ± 0.38	1.2
Fructose 6-phosphate	0.38 ± 0.06	0.59 ± 0.08	1.5*
Fructose 1,6-bisphosphate	17.53 ± 0.82	20.52 ± 0.66	1.2*
Glyceraldehyde 3-phosphate	0.15 ± 0.01	0.19 ± 0.01	1.3*
Dihydroxyacetone phosphate	0.79 ± 0.13	2.08 ± 0.66	2.6
3-Phosphoglycerate	1.30 ± 0.12	2.52 ± 0.28	1.9*
2-Phosphoglycerate	0.16 ± 0.01	0.17 ± 0.01	1.0
Phosphoenolpyruvate	0.25 ± 0.01	0.12 ± 0.01	0.5*
Trehalose synthesis			
Trehalose 6-phosphate	9.34 ± 1.18	14.48 ± 1.67	1.6*
Trehalose	0.20 ± 0.04	0.41 ± 0.09	2.1*
Pentose phosphate pathway			
6-Phosphoglucononate	1.01 ± 0.02	1.11 ± 0.07	1.1
Ribose 5-phosphate	0.40 ± 0.02	0.51 ± 0.03	1.3*
Ribulose 5-phosphate	0.20 ± 0.03	0.33 ± 0.06	1.6*
Xylulose 5-phosphate	0.43 ± 0.07	0.72 ± 0.13	1.7
Sedoheptulose 7-phosphate	0.50 ± 0.06	0.54 ± 0.04	1.1
Erythrose 4-phosphate	0.004 ± 0.000	0.005 ± 0.000	1.3*
TCA cycle and glyoxylate cycle			
Citrate	4.56 ± 0.65	5.80 ± 0.98	1.3
Isocitrate	0.02 ± 0.00	0.03 ± 0.00	1.7*
α-Ketoglutarate	0.29 ± 0.02	0.67 ± 0.24	2.3
Succinate	0.37 ± 0.03	0.37 ± 0.12	1.0
Fumarate	0.10 ± 0.02	0.18 ± 0.06	1.8
Malate	0.60 ± 0.08	1.03 ± 0.19	1.7*
Nucleotides and cofactors			
Energy charge	0.86 ± 0.00	0.88 ± 0.01	1.0
AMP	0.13 ± 0.01	0.17 ± 0.01	1.3*
ADP	1.55 ± 0.08	1.43 ± 0.11	0.9
ATP	5.50 ± 0.16	5.58 ± 0.21	1.0
UDP	0.23 ± 0.01	0.22 ± 0.05	0.9
UTP	1.61 ± 0.06	1.57 ± 0.10	1.0
GDP	0.27 ± 0.02	0.21 ± 0.07	0.8
GTP	1.58 ± 0.06	1.49 ± 0.15	0.9
CDP	0.13 ± 0.02	0.15 ± 0.01	1.1
CTP	0.84 ± 0.02	0.82 ± 0.03	1.0
NADH	0.18 ± 0.02	0.15 ± 0.02	0.8
NAD^+^	85.43 ± 1.90	91.82 ± 2.64	1.1*
NADP^+^	8.49 ± 0.67	6.87 ± 0.25	0.8*
Acetyl-CoA	5.80 ± 0.18	5.38 ± 0.34	0.9
FAD^+^	0.87 ± 0.12	0.54 ± 0.18	0.6

a Intracellular metabolite contents were measured during the mid-exponential glucose phase of aerobic bioreactor batch cultures of S. cerevisiae CEN.PK113-7D (naive reference) and IMX2538 (minimal CCM) (see Table 2 for other physiological data). Data represent averages and standard deviations of data from analyses of three independent cultures for each strain. Fold differences that are statistically significant are indicated in bold with an asterisk (two-tailed *t* test, equal variances, *P* < 0.05).

### Dissecting individual from synergistic responses to growth under a range of conditions.

To explore genetic redundancy of CCM genes, the minimal CCM strain and the congenic reference strain CEN.PK113-7D were grown under a broad range of conditions. Some of these were chosen based on previously reported phenotypes (e.g., high osmolarity) or connection to CCM (e.g., growth on various carbon sources), while others subjected the strains to adverse conditions (e.g., acidic or alkaline pH).

**FIG 4:**
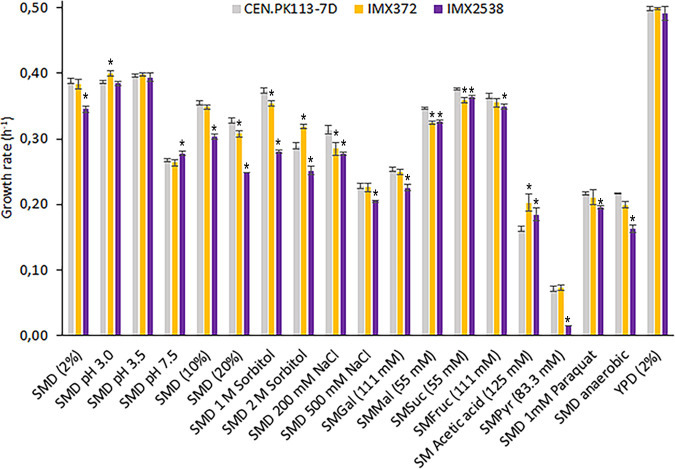
Specific growth rates of the 35-deletion, prototrophic minimal CCM strain under a broad range of growth conditions. Specific growth rates of the prototrophic S. cerevisiae strains CEN.PK113-7D (naive reference strain), IMX372 (minimal glycolysis (MG)), and IMX2538 (minimal CCM) under different growth conditions are shown. Specific growth rates were measured in triplicate cultures using a growth profiler, except for those in the SMPyr and SMD-anaerobic cultures, which were measured in independent duplicate shake flask cultures. Abbreviations indicate the following growth conditions: SM, synthetic medium; SMD, synthetic medium with glucose; Gal, galactose as carbon source; Mal, maltose as carbon source; Suc, sucrose as carbon source; Fruc, fructose as carbon source; Pyr, pyruvate as carbon source; YPD, complex medium with glucose. Significant changes in growth rates of IMX372 and IMX2538 with respect to that of CEN.PK113-7D are indicated with an asterisk (*P* < 0.05, two-tailed paired homoscedastic *t* test).

Consistent with reports that deletion of *GPD1* causes decreased osmotolerance (123, 125), the minimal CCM strain grew 13% to 25% slower than the reference strain exposed to high osmolarity, which was imposed by adding high concentrations of sorbitol (1 M and 2 M) or glucose (10% and 20% [wt/vol]). Construction and analysis of strains with different combinations of deletions in reference and CCM minimization backgrounds confirmed that this growth reduction specifically resulted from *GPD1* deletion, rather than from synergistic effects (Fig. 4; see also Fig. S3). *MPC3* deletion has been reported to cause lower growth rates on glycerol or lactate as sole carbon source (77). Since Mpc3 is a pyruvate transporter, growth was assessed directly on chemically defined medium with pyruvate as sole carbon source. The minimal CCM strain grew 79% slower than the control strain (Fig. 4); however, this could not be attributed to the *MPC3* deletion, surprisingly. Indeed, reintroduction of *MPC3* in IMX2519 (CCMin 5, glyc^min^ fer^min^ ppp^min^ tca^min^ mc^min^ fum^min^ glyox^min^ Ace^min^ glycerol^min^), resulting in strain IMX2641, did not restore the growth rate on pyruvate (see Fig. S3G).

10.1128/mBio.02970-21.3 FIG S3 Growth rates in specific environments. (A to D) Specific growth rate determinations under osmotic stress conditions (supplemented with uracil), measured with the growth profiler in biological triplicates. (E) Specific growth rates of *FRDS1* and *AAC3* deletion mutants on SMD supplemented with Tween, ergosterol, and uracil measured under anaerobic conditions in shake flasks in biological duplicates. (F) Specific growth rates of *MPC3* complementation strains on SME supplemented with uracil in shake flasks in biological duplicates. (G) Specific growth rate of *MPC3* complementation strain on SM with 83.3 mM pyruvate and supplemented with uracil in shake flasks in biological duplicates. Significant changes in growth rates with respect to the parental strain are indicated (*, *P* < 0.05, two-tailed paired homoscedastic *t* test). Download FIG S3, PDF file, 0.2 MB. Copyright © 2022 Postma et al. 2022 Postma et al. https://creativecommons.org/licenses/by/4.0/ This content is distributed under the terms of the Creative Commons Attribution 4.0 International license.

Combined deletion of *AAC1*, *AAC3*, *MPC3*, and *SAL1* caused a 3 to 5% and a 14 to 18% decrease of growth rates on SMD and SME, respectively, thus marking their importance on these carbon sources. According to previous reports, individual deletion of these four genes does not affect growth on glucose and individual deletion of *AAC1*, *AAC3*, and *SAL1* does not affect growth on ethanol (64, 65, 77, 131). Deletion of *MPC3* has been reported to cause a decrease in growth rate on glycerol and lactate (77) and may therefore also be responsible for the lower growth rate on ethanol. Reintroduction of *MPC3* in strain IMX1984 (glyc^min^ fer^min^ ppp^min^ tca^min^ *aac1Δ aac3Δ sal1Δ mpc3Δ*) increased the specific growth rate on SME by only 3%, while reintroduction in IMX2519 (CCMin 5, glyc^min^ fer^min^ ppp^min^ tca^min^ mc^min^ fum^min^ glyox^min^ ace^min^ glycerol^min^) did not affect growth rate (see Fig. S3). These results suggest that the observed impact of quadruple deletion of *AAC1*, *AAC3*, *MPC3*, and *SAL1* on ethanol growth was caused by synergistic effects.

Some paralogs have been reported to be specifically important under anaerobic conditions (*AAC3*, *FRDS1*) (94, 131). In line with these reports, while the MG strain showed the same growth rate as CEN.PK113-7D under anaerobic conditions, the minimal CCM strain showed a 25% lower anaerobic growth rate (Fig. 4). However, testing of deletions in the reference background indicated that this difference was not caused by the deletion of *AAC3* or *FRDS1* (see Fig. S3).

Over the broad range of conditions tested, including several stress conditions, few differences in specific growth rate were observed (Fig. 4). Although lag phases were observed for some conditions, as expected from cells transitioning between different growth environments (the inoculum was consistently prepared using SM glucose as medium and under standard conditions regarding pH, temperature, etc.), no difference in lag phase was observed between the final IMX2519 mutant and the control strain. Combining the phenotypes of strains with individual and clustered deletions enabled us to identify synergistic interactions between minor paralogs.

## DISCUSSION

Genetic reduction has been applied in several microorganisms (1), including Bacillus subtilis (3), Escherichia coli (2), Lactococcus lactis (6), Corynebacterium glutamicum (4), *Streptomyces* species (7), Pseudomonas species (5) and Schizosaccharomyces pombe (132), with the purpose of discovering a minimal genome content and/or for engineering efficient cell factories. In S. cerevisiae, Marakami et al. reduced genome content by 5% by deleting 15 terminal chromosomal regions (133). Moreover, the creation of a synthetic yeast genome in the Sc2.0 project was accompanied by an 8% genome reduction by deletion of long terminal repeats, retrotransposons, and introns; in addition, engineering of a single-chromosome yeast strain was characterized by a 9% decrease in DNA (134, 135). Genome reduction studies typically target two types of DNA sequences, nonexpressed DNA (cryptic genes, mobile DNA) and irrelevant or nonessential genes. These DNA elements can be targeted by random strategies for which little knowledge is required, such as transposon mutagenesis or the elegant SCRaMbLE technique used for the recent reduction of the left synthetic chromosome arm XII in S. cerevisiae (21). In the present study, knowledge-based reduction of the gene complement for CCM in S. cerevisiae was informed by gene expression data and previous phenotypic analyses on single-knockout mutants (21, 133–135).

In this study, we reduced genetic complexity of CCM in S. cerevisiae by deletion of 35 genes encoding enzymes and transporters. This reduction corresponded to elimination of 32% of the (iso)enzymes and transporters involved in the included processes, without major impacts on strain physiology, which was tested under a broad range of conditions (Fig. 2 and 4). The present study built on earlier work by Solis-Escalante et al. (22), who eliminated 50% of isoenzymes involved in glycolysis and ethanolic fermentation with a similar small impact on physiology. The attainable reduction of gene sets for enzymes and transporters involved in other CCM pathways differed, with 50% for fumarate reduction and glycerol synthesis, 37% for the mitochondrial carriers, 36% for the pentose-phosphate pathway, 23% for the TCA cycle, 14% for the glyoxylate cycle, 20% for the glycolysis-TCA cycle interface plus gluconeogenesis, and 8% for acetyl-CoA metabolism. The lower attainable genetic reduction of the four latter pathways can be largely attributed to neofunctionalization and relocalization of enzymes during evolution.

Our results showed that yeast CCM is remarkably robust to genetic reduction, in particular during growth on glucose, yeast’s favorite carbon source, but also when challenged by a broad range of growth conditions. Notable exceptions were growth on pyruvate (79% growth rate reduction), anaerobic growth on glucose (25% reduction), growth on ethanol (24% reduction), and growth at high osmolarity (between 13% and 25% lower specific growth rates). Growth rate reductions on ethanol and at high osmolarity could be attributed to specific genes or gene combinations, while for growth on pyruvate and anaerobic growth, some hypothetical targets could be excluded. The physiological role of most deleted paralogs remains elusive. Such a situation is exemplified by *TKL2* and *NQM1*, which are paralogs of the major PPP genes *TKL1* and *TAL1*, respectively. In S. cerevisiae strains engineered for l-arabinose utilization, their deletion was shown to lead to lower growth rates on this pentose sugar (33, 136). Clearly, as pentoses are not natural carbon sources for S. cerevisiae, this role cannot have provided an evolutionary driving force for fixation of these paralogs in its genome, but it does indicate potential contributions to fitness under other, as-yet-unidentified growth conditions. Testing the minimal CCM strain under an even wider variety of environmental conditions, including dynamics in nutrient availability and other environmental parameters, may reveal physiological roles of these and other paralogs. Alternatively, the mechanisms that fixed some paralogs during evolution may have been disrupted by relatively recent mutations or gene loss (137). Following this reasoning, absence of a clear phenotype of knockout mutants may have captured a stage in the evolutionary trajectory of S. cerevisiae that will eventually lead to loss of a paralog, evolution toward complete subfunctionalization, or retention of functional overlap with asymmetric divergence (138).

In this first step toward the genetic minimization of CCM in yeast, choices had to be made on which pathways and genes were considered part of the CCM and on criteria for redundancy. For instance, transport of NAD^+^, FAD^+^, ADP/ATP, and P_i_ across the mitochondrial membrane was considered, while transport of NAD(P)H, which requires more complex shuttle systems (139, 140), was not yet included. In addition, as S. cerevisiae cannot synthetize carnitine (141), the carnitine shuttle system transporting acetyl-CoA across compartments was not considered. Since *CRC1*, *CAT1*, *YAT1*, and *YAT2* involved in this shuttle are dispensable for growth in the absence of carnitine (141), they can be considered for further genetic reduction of the CCM. Some genes required for anaerobic growth, such as *ADH3* (139, 142), were also retained but could be removed if fast anaerobic growth is excluded as a criterion. Several other processes and pathways are of particular interest for development of strain platforms for modular engineering of yeast CCM. In this context, glucose uptake, which involves a set of 20 hexose transporters (143), provides an interesting target for future experiments, whose minimization can benefit from a recently constructed Hxt^0^ CRISPR kit (144). Another logical target for minimization is uptake and assimilation of (alternative) carbon sources and especially of maltose, whose metabolism is enabled by highly redundant subtelomeric genes (145, 146).

Genetic reduction presents a first, indispensable step toward the construction of modular yeast strains for extensive remodeling of CCM. Current demands for economically competitive cell factories, with optimized titer, rate, and yield, requires extensive remodeling of the CCM for the supply of precursors, (redox) cofactors and energy-rich molecules (147–149). For instance, the extensive remodeling of the native Entner-Doudoroff glycolytic pathway into the Embden-Meyerhof-Parnas pathway improved carotenoid synthesis in Pseudomonas putida (150). Similarly, substantial efforts have been invested in remodeling yeast CCM in S. cerevisiae to increase the supply of cytosolic acetyl-CoA, a precursor for a wide array of attractive biomolecules (Fig. 1) (151). Also, production of complicated chemical structures, like plant natural products in S. cerevisiae, requires extensive remodeling of the entire central carbon metabolism (152–154). As demonstrated by Kuijpers and coworkers (24), genetic reduction facilitates the colocalization of sets of genes in “pathway clusters” and strongly accelerates the genetic remodeling of these pathways. With this strategy, the 12 steps of glycolysis and ethanolic fermentation were rapidly and efficiently swapped with heterologous variants and enabled the implementation of an innocuous DNA and RNA watermarking method (155). A similar strategy can be considered for remodeling CCM, with the minimal CCM strain as starting point. As recently demonstrated, 44 transcriptional unit-sized DNA fragments can be assembled in S. cerevisiae into specialized, synthetic supernumerary chromosomes (26). Since the capacity of homologous recombination was not reached, assembly of synthetic chromosomes containing the set of 76 genes encoding the minimal CCM has now become a realistic objective. Subsequent CRISPR-Cas-assisted removal of the duplicate CCM genes from their native locations could then generate powerful platforms for chromosome swapping and combinatorial CCM remodeling studies. The reduction of genetic complexity demonstrated in the present study therefore not only provides new insights in genetic redundancy of CCM but also contributes to the eventual localization of all genes required for a minimized CCM on specialized, synthetic supernumerary chromosomes that allow for extensive, combinatorial remodeling of yeast metabolism for industrial applications.

## MATERIALS AND METHODS

### Strains, media and maintenance.

The Saccharomyces cerevisiae strains used in this study were all derived from the CEN.PK family (156, 157) (see Table S3). The naive, uracil-auxotrophic, and Cas9-containing strain, IMX581 (158), and the uracil-auxotrophic MG (minimal glycolysis) strain IMX370 (22) were used for deletion of genes encoding enzymes or transporters involved in CCM. The naive uracil-prototrophic strain, CEN.PK113-7D, was used for physiological comparison. Complex medium used for propagation of yeast strains consisted of 10 g liter^−1^ Bacto yeast extract, 20 g liter^−1^ Bacto peptone, and 20 g liter^−1^ glucose (YPD), autoclaved at 110°C for 20 min. After transformation, yeast strains were selected in synthetic medium (SM) (159) containing 3.0 g liter^−1^ KH_2_PO_4_, 0.5 g liter^−1^ MgSO_4_·7H_2_O, 5.0 g liter^−1^ (NH_4_)_2_SO_4_, and 1.0 mL liter^−1^ trace elements autoclaved at 121°C for 20 min, whereafter 1.0 mL liter^−1^ of filter-sterilized vitamin solution was added. Before autoclaving, media were set to pH 6 by 1 M KOH addition. SM was supplemented with 20 g liter^−1^ glucose (SMD) or 2% ethanol (vol/vol) (SME) for propagation and growth characterization. Synthetic medium was supplemented with 150 mg liter^−1^ uracil for uracil-auxotrophic strains. For selection of transformants carrying the amdS selection marker (160), ammonium sulfate was replaced as nitrogen source with 10 mM acetamide. For experiments on SM in the growth profiler and under anaerobic conditions, ammonium sulfate was replaced by 2.3 g liter^−1^ urea. For both media in which ammonium sulfate was replaced, 6.6 g liter^−1^ K_2_SO_4_ was added. Growth was performed in 500-mL shake flasks containing 100 mL medium or in 100-mL shake flasks containing 20 mL medium at 30°C and 200 rpm in an Innova 44 incubator shaker (New Brunswick Scientific, Edison, NJ). Cultures on solid media were incubated for 3 to 5 days at 30°C.

10.1128/mBio.02970-21.6 TABLE S3 Strains. Download Table S3, PDF file, 0.2 MB. Copyright © 2022 Postma et al. 2022 Postma et al. https://creativecommons.org/licenses/by/4.0/ This content is distributed under the terms of the Creative Commons Attribution 4.0 International license.

CEN.PK113-7D, IMX372, IMX2538, and several intermediate strains were tested in the growth profiler on a variety of liquid media containing SM (urea) plus 2% glucose (SMD), SMD at pH 3.0, 3.5, or 7.5, SM plus 10% glucose (SMD [10%]), SM plus 20% glucose (SMD [20%]), SMD plus 1 or 2 M sorbitol, SMD plus 200 or 500 mM NaCl, SM plus 111 mM galactose (SMGal), SM plus 55 mM maltose (SMMal), SM plus 55 mM sucrose (SMSuc), SM plus 111 mM fructose (SMFruc), SM plus 125 mM acetic acid, SMD plus 1 mM paraquat and YPD (2% glucose). Growth on SM plus 83.3 mM pyruvic acid was performed in shake flasks. For anaerobic growth in shake flasks, SMD was supplemented with 0.01 g liter^−1^ ergosterol and 0.42 g liter^−1^ Tween 80 dissolved in ethanol (SMD [2%] anaerobic) (159).

Plasmids were propagated in and isolated from chemically competent Escherichia coli XL1-Blue cells, which were cultivated in lysogeny broth containing 10 g liter^−1^ Bacto tryptone, 5.0 g liter^−1^ Bacto yeast extract, and 5 g liter^−1^ NaCl supplemented with 100 mg liter^−1^ ampicillin (LB-amp) when required. E. coli was cultivated in 15-mL Greiner tubes containing 5 mL medium at 37°C and 200 rpm in an Innova 4000 incubator shaker (New Brunswick Scientific). Bacterial cultures on solid medium were incubated overnight at 37°C.

For solid medium, 20 g liter^−1^ of agar was added before autoclaving. All S. cerevisiae and E. coli strains were stored at −80°C in 1-mL aliquots containing 30% (vol/vol) glycerol in appropriate medium.

### Molecular biology techniques.

Plasmids were isolated from E. coli using the GenElute plasmid miniprep kit (Sigma-Aldrich, St. Louis, MO) or the GeneJET plasmid miniprep kit (Thermo Fisher Scientific, Waltham, MA) according to the provided protocols. DNA fragments for plasmid construction or integrative DNA fragments used in yeast transformation were amplified using Phusion high-fidelity DNA polymerase (Thermo Fisher Scientific) according to the manufacturer’s instructions, using PAGE-purified or desalted oligonucleotides (Sigma-Aldrich) depending on the application. Purification of genomic PCR-amplified DNA was performed with the GenElute PCR Clean-Up kit (Sigma-Aldrich) or the GeneJET PCR purification kit (Thermo Fisher Scientific) if no aspecific products were present. When aspecific products were present or when DNA was amplified from plasmids, the DNA was purified by separation using electrophoresis on a 1% (wt/vol) agarose gel (TopVision agarose, Thermo Fisher Scientific) in 1× Tris-acetate-EDTA buffer (Thermo Fisher Scientific) or on a 2% (wt/vol) agarose gel (TopVision agarose) in 1× Tris-borate-EDTA buffer (Thermo Fisher Scientific) with subsequent purification with the Zymoclean gel DNA recovery kit (Zymo Research). Chemical transformation of E. coli XL1-Blue was performed by thawing of competent cells on ice, addition of DNA, and heat shock for 40 s at 42°C. Subsequently, cells were incubated on ice for 2 min and plated immediately on selective LB-amp plates and grown overnight at 37°C. Transformation of S. cerevisiae was performed using the lithium acetate–single-stranded carrier DNA–polyethylene glycol method (161). Single-cell lines were obtained by three consecutive restreaks on selective solid medium. Yeast genomic DNA was extracted according to the methods of Looke et al. (162), with the YeaStar genomic DNA kit (Zymo Research, Irvine, CA) according to Protocol I supplied by the manufacturer, or the Qiagen blood and cell culture kit with 100-G or 20-G genomic tips (Qiagen, Hilden, Germany) following the manufacturer’s recommendations. Verification of the accurate genotypes of engineered S. cerevisiae strains and E. coli plasmids was done by diagnostic PCR before strain storage at −80°C. These diagnostic PCRs were performed using desalted oligonucleotides and the DreamTaq PCR master mix (Thermo Fisher Scientific) according to the manufacturer’s protocol.

### Plasmid and strain construction.

Deletions were performed using CRISPR/Cas9. CRISPR/Cas9-based genome editing of S. cerevisiae was performed as described by Mans et al.(158) with minor alterations. Plasmids containing a single guide RNA (gRNA) (see Table S4) were constructed via Gibson assembly with a backbone containing the marker cassette and one insert fragment containing the gRNA and the 2μm plasmid fragment. The backbone was amplified from a pMEL plasmid (158) with primers 5980 and 5792, and the insert fragment was amplified with a gRNA-specific primer designed with the yeastriction tool (158) and primer 5979 (primers are listed in Table S5). Plasmids containing two gRNAs were constructed using one backbone fragment and two insert fragments, each containing one gRNA and one half of the 2μm fragment. Backbones were PCR amplified from the pROS plasmids (158) with the double-binding primer 6005. Insert fragments were obtained with the gRNA-specific primers together with either primer 5974 or primer 5975 (see Table S5). The backbone and gRNA insert fragment(s) were gel purified, DpnI digested (Thermo Fisher Scientific), and Gibson assembled in a final volume of 5 μL using NEBuilder HiFi DNA assembly master mix (NEB, Ipswich, MA), according to the manufacturer’s instructions. Assembled plasmids were transformed and subsequently isolated from LB-amp-grown E. coli. Correct assembly was checked using diagnostic PCR (see Table S5).

10.1128/mBio.02970-21.7 TABLE S4 Plasmids. Download Table S4, PDF file, 0.2 MB. Copyright © 2022 Postma et al. 2022 Postma et al. https://creativecommons.org/licenses/by/4.0/ This content is distributed under the terms of the Creative Commons Attribution 4.0 International license.

10.1128/mBio.02970-21.8 TABLE S5 Primers used in this study. Download Table S5, PDF file, 0.2 MB. Copyright © 2022 Postma et al. 2022 Postma et al. https://creativecommons.org/licenses/by/4.0/ This content is distributed under the terms of the Creative Commons Attribution 4.0 International license.

IMK588 was constructed by integrating the *KanMX* marker at the *OAC1* locus of CEN.PK113-7D. The *KanMX* marker with homologous flanks to *OAC1* was amplified with primers 6358 and 6359 from pUG6 (see Tables S4 and S5).

In order to perform CRISPR editing in the MG strain (IMX370), *Cas9* was integrated by transforming a *Cas9* and *natNT2* DNA fragment, which could assemble by homologous recombination at the *CAN1* locus. The *Cas9* fragment (*can1* flank-*Cas9* expression cassette-SHR A) was PCR amplified with primers 2873 and 4653 from plasmid p414-*TEF1p-Cas9-CYC1t* (see Tables S4 and S5). The *natNT2* fragment (SHR A-NatNT2 marker cassette-*can* 1 flank) was amplified with primers 3093 and 5542 from plasmid pUG-*natNT2* (see Tables S4 and S5). The *Cas9* containing MG strain was stocked as IMX1331.

For genome editing using CRISPR, S. cerevisiae strains were transformed with 1 μg of each gRNA plasmid and 1 μg of each 120-bp double-stranded DNA repair fragment. These repair fragments were made by annealing of complimentary oligonucleotides listed in Table S5 and consisted of 60-bp homology sequences immediately upstream of the start codon and downstream of the stop codon of the targeted gene, unless stated otherwise. Transformants were plated on selective medium. Gene deletion was verified by diagnostic colony PCR on randomly picked colonies by using the primers which bind outside of the targeted open reading frame (ORF) (see Table S5). gRNA plasmids were removed by growing the colonies in liquid YPD medium and subsequent plating on solid YPD medium. Plasmid removal was confirmed by growth on selective and nonselective solid media after which the strains were stored. For all transformations, the corresponding gRNA plasmids and repair fragment are summarized in Table S6.

10.1128/mBio.02970-21.9 TABLE S6 Strain transformations. Download Table S6, PDF file, 0.2 MB. Copyright © 2022 Postma et al. 2022 Postma et al. https://creativecommons.org/licenses/by/4.0/ This content is distributed under the terms of the Creative Commons Attribution 4.0 International license.

To obtain a prototrophic strain with 35 deletions, a *URA3* transcriptional unit amplified from CEN.PK113-7D DNA (primers 17752 and 17753 [see Table S5]) was integrated at the *GPP2* locus of strain IMX2520 (34 deletions). The flanks of the *URA3* repair fragment were homologous to the 60 bp immediately upstream and downstream of the *GPP2* ORF. The prototrophic 35-deletion strain IMX2538 was checked by diagnostic PCR, short-read sequencing, and long-read nanopore sequencing. Integration of the *MPC3* transcriptional unit at the *X2* locus of IMX1984 and IMX2519 was achieved by amplifying the corresponding fragment from CEN.PK113-7D genomic DNA (primers 18025 and 18026 [see Table S5]) and integration by CRISPR-Cas9 using gRNA plasmid pUDR376, resulting in strains IMX2640 and IMX2641, respectively. Correct integration was verified by diagnostic PCR (see Table S5).

### Sequencing.

High-quality genomic DNA of yeast for sequencing was extracted using the Qiagen blood and cell culture kit with 100-G or 20-G genomic tips (Qiagen) according to the manufacturer’s instructions. DNA concentration was measured using the BR ds DNA kit (Invitrogen, Carlsbad, CA) and a Qubit 2.0 fluorometer (Thermo Fisher Scientific). The purity was verified with a Nanodrop 2000 UV-Vis spectrophotometer (Thermo Fisher Scientific).

### Short-read sequencing.

IMX2538 (35-deletions prototrophic strain) was sequenced using 300-bp paired-end sequencing reads prepared with the MiSeq reagent kit v3 on an Illumina MiSeq sequencer (Illumina, San Diego, CA). To this end, extracted DNA was mechanically sheared to 550 bp with the M220 ultrasonicator (Covaris, Wolburn, MA) and subsequently the TruSeq DNA PCR-Free Library preparation kit (Illumina) was employed to make a six-strain library. The samples were quantified by quantitative PCR on a Rotor-Gene Q PCR cycler (Qiagen) using the KAPA library quantification kit (Kapa Biosystems, Wilmington, MA). Library integrity and fragment size were determined with a Tapestation 2200 system (Agilent Technologies). Sequencing reads were mapped onto the CEN.PK113-7D (163) reference genome using the Burrows-Wheeler alignment (BWA) tool (version 0.7.15) (164) and further processed using SAMtools (version 1.3.1) (165) and Pilon (with -vcf setting; version 1.18) (166) to identify SNPs. The sequence was analyzed by visualizing the generated .bam files in the Integrative Genomics Viewer (IGV) software (version 2.4.0) (167). Chromosomal copy number was estimated by the Magnolya algorithm (version 0.15) (168).

### Long-read sequencing.

High-quality DNA of IMX2538 was isolated and checked for quantity and quality as described above. Furthermore, quality and integrity of DNA was checked with a TapeStation 2200 system (Agilent Technologies, Santa Clara, CA). IMX2538 was sequenced in-house on a single R10 flow cell (FLO-MIN111) using the SQK-LSK109 sequencing kit (Oxford Nanopore Technologies, Oxford, United Kingdom), according to the manufacturer’s instructions. With MinKnow (version 3.6.5, Oxford Nanopore Technologies), raw signal files were generated. Base-calling was performed by Guppy (version 4.0.11, Oxford Nanopore Technologies), followed by *de novo* assembly with Canu (version 2.0) (169).

### Growth rate measurements in shake flasks.

The growth rate of the constructed strains was determined in 500-mL shake flasks containing 100 mL of SMD or SME medium. Wake-up cultures were inoculated with a 1-mL aliquot of a strain stored at −80°C and grown until late exponential phase. Precultures were inoculated from the wake-up cultures and grown to mid-exponential phase. Finally, measuring cultures were inoculated in biological duplicates from the preculture at an initial optical density at 660 nm (OD_660_) of 0.3. Cultures were monitored based on the OD_660_ with a Jenway 7200 spectrophotometer in technical duplicate (Cole-Parmer, Vernon Hills, IL). A maximum specific growth rate (μ_max_) was calculated from at least five data points in the exponential phase with at least 2 doublings.

Anaerobic shake flask-based experiments were performed at 30°C in a Bactron anaerobic chamber (Sheldon Manufacturing Inc., Cornelius, OR) with an atmosphere of 5% (vol/vol) H_2_, 6% (vol/vol) CO_2_, and 89% (vol/vol) N_2_, on an IKA KS 260 basic shaker at 200 rpm, using 50-mL shake flasks containing 30 mL SMD (2%) anaerobic medium.

### Growth rate measurements in microtiter plates.

Growth measurements of strains in microtiter plates with a Growth Profiler 960 system (EnzyScreen BV, Heemstede, The Netherlands) were performed as described by Postma et al. (26). Briefly, strains from glycerol freezer stocks were inoculated and grown overnight in 10 mL SMD medium in a 50-mL shake flask. This culture was used to inoculate a preculture in 10 mL SMD medium in a 50-mL shake flask, which was cultivated until mid-exponential growth. To prepare the inoculum, the cells were then spun down and resuspended in SM without carbon source. The growth study was performed in 96-well microtiter plates (EnzyScreen type CR1496dl), with a final working volumes of 250 μL and an approximate starting OD_660_ of 0.3. Microtiter plates were closed with a sandwich cover (EnzyScreen type CR1296). Images of cultures were made at 30-min intervals. Growth rates were calculated in the time frame where the calculated OD was between 2 and 10. Each experimental condition was analyzed in biological triplicates.

### Physiological characterization of CEN.PK113-7D and IMX2538 in bioreactor cultures.

Aerobic batch bioreactor cultures were performed in 2-liter bioreactors (Applikon, Delft, The Netherlands). Bioreactors were filled with synthetic medium containing 5.0 g liter^−1^ (NH_4_)_2_SO_4_, 3.0 g liter^−1^ KH_2_PO_4_, 0.5 g liter^−1^ MgSO_4_·7H_2_O, and 1.0 mL liter^−1^ trace elements. After heat sterilization, 20 g liter^−1^ glucose, 0.2 g liter^−1^ antifoam emulsion C (Sigma-Aldrich, St. Louis, MA), and filter-sterilized vitamins were added to complete the medium. Upon inoculation, bioreactors contained a working volume 1.4 liters and the culture pH was maintained at 5.0 by automated addition of 2 M H_2_SO_4_ or 2 M KOH. Temperature was kept stable at 30°C, and mixing of the medium was performed at 800 rpm. The gas flow was set to 700 mL of air per minute to supply oxygen and remove produced carbon dioxide, and an overpressure of 0.3 × 10^5^ Pa was applied to the reactor. Dissolved oxygen tension was thus maintained for all reactors above 59% over the whole duration of the batch cultivation. Off-gas was cooled to 2°C in a condenser on the bioreactor to prevent water evaporation and further dried with a Permapure MD-110-48P-4 filter dryer (Permapure, Lakewood, NJ) for subsequent analysis of carbon dioxide and oxygen percentages by a MultiExact 4100 gas analyzer (Servomex, Zoetermeer, The Netherlands). For both CEN.PK113-7D and IMX2538, reactors were run in biological triplicates and inoculated from exponentially growing shake flask cultures.

Optical densities were measured in technical triplicates with a Jenway 7200 spectrophotometer (Cole-Parmer) at 660 nm, while cell dry weights were determined by filtration of 10 mL of well-mixed sample over dried polyethersulfone (PES) membrane filters with a pore size of 0.45 μm (Pall Corporation, Port Washington, NY). Filters were washed with demineralized water and dried in a microwave oven for 20 min at 360 W.

Extracellular organic acids, sugars, and ethanol were determined by high-performance liquid chromatography analysis using an Aminex HPX-87H ion-exchange column (Agilent, Santa Clara) with 5 mM H_2_SO_4_ as mobile phase and a flow rate of 0.6 mL min^−1^ at 60°C. Glucose, glycerol, and ethanol were detected by a refractive index detector (Agilent G1362A), and organic acids were detected by a dual-wavelength absorbance detector (Agilent G1314F).

During mid-exponential growth in the glucose consumption phase, intracellular metabolite samples were taken with a filtration-based washing method according to that of Douma et al. (170) with some modifications. Briefly, approximately 3 mL of cell culture was sampled in 15 mL of 100% methanol at −40°C. Biomass was washed with cooled 100% methanol on a PES membrane with a pore size of 0.45 μm (Pall Corporation), which was precooled and wetted with 100% methanol at −40°C. Finally, metabolites were extracted with 75% boiling ethanol. A 100-μL volume of ^13^C-labeled cell extract was added to each tube as an internal standard for metabolite quantification (171). The intracellular CCM metabolites, cofactors, and nucleotides were derivatized and quantified as described by de Jonge et al. (172) and Niedenführ et al. (173).

### Data availability.

Short- and long-read sequencing data are available at NCBI under BioProject PRJNA757356.
