# Supplementary material for: Top-Down, Knowledge-Based Genetic Reduction of Yeast Central Carbon Metabolism
Source: mBio. 2022 Sep 21;13(5):e02970-21. doi: 10.1128/mbio.02970-21 (PMC9600970; doi:10.1128/mbio.02970-21)
Supplement: TABLE S1 [file mbio.02970-21-s0004.pdf]

Table S1 Genetic characteristics of the paralogs considered for deletion

Genes that were deleted in this study are marked in red. Protein similarity was based on global alignment using BLOSUM62 of the translated genes of the *S. cerevisiae* CEN.PK113-7D genome sequence (Salazar *et al.* 2017). WGD pairs are indicated (Byrne and Wolfe 2005).

Pathway	Gene	% Protein Similarity	Type of duplication
**Pentose phosphate pathway**	*GND1* (YHR183W) / *GND2* (YGR256W)	87%	WGD
	TKL1 (YPR074C) / *TKL2* (YBR117C)	71%	WGD
	SOL3 (YHR163W) / *SOL4* (YGR248W)	47%	WGD
	TAL1 (YLR354C) / *NQM1* (YGR043C)	68%	WGD
**TCA cycle + anaplerotic reactions**	*PYC1* (YGL062W) / *PYC2* (YBR218C)	92%	WGD
	SDH1 (YKL148C) / *SDH1b* (YJL045W)	84%	WGD
	SDH3 (YKL141W) / *SHH3* (YMR118C)	42%	WGD
	SDH4 (YDR178W) / *SHH4* (YLR164W)	47%	WGD
	CIT1 (YNR001C) / *CIT2* (YCR005C) / *CIT3* (YPR001W)	CIT1/CIT2=75% **CIT1/CIT3=42%**	CIT1/CIT2=WGD
**Mitochondrial transporters**	*AAC1* (YMR056C)/ *AAC2* (YBL030C) / *AAC3* (YBR085W)	AAC2/AAC1=73% AAC2/AAC3=87%	AAC2/AAC3=WGD
	SAL1 (YNL083W)		
	MPC1 (YGL080W) / *MPC2* (YHR162W) / *MPC3* (YGR243W)	**MPC2/MPC3=65%** MPC2/MPC1=25%	MPC2/MPC3=WGD
	CTP1 (YBR291C)		
	ODC1 (YPL134C) / *ODC2* (YOR222W)	61%	WGD
	NDT1 (YIL006W) / *NDT2* (YEL006W)	51%	WGD
**Fumarate reductase**	*OSM1* (YJR051W) / *FRDS1* (YEL047C)	59%	WGD
**Glyoxylate and TCA cycle**	*IDP1* (YDL066W) / *IDP2* (YLR174W) / *IDP3* (YNL009W)	IDP1/IDP3=65% IDP2/IDP3 =73%	IDP2/IDP3=WGD

**acetyl-CoA synthesis**	*ALD2* (YMR170C) / *ALD3* (YMR169C)	91%	
**Glycerol synthesis**	*GPD1* (YDL022W) / *GPD2* (YOL059W)	72%	WGD
	GPP1 (YIL053W) / *GPP2* (YER062C)	92%	WGD

Salazar AN, Gorter de Vries AR, van den Broek M et al. Nanopore sequencing enables near-complete *de novo* assembly of *Saccharomyces cerevisiae* reference strain CEN.PK113-7D. FEMS Yeast Res 2017;17.

Byrne KP, Wolfe KH. The Yeast Gene Order Browser: combining curated homology and syntenic context reveals gene fate in polyploid species. Genome Res 2005;15: 1456-61.
