# Supplementary material for: Top-Down, Knowledge-Based Genetic Reduction of Yeast Central Carbon Metabolism
Source: mBio. 2022 Sep 21;13(5):e02970-21. doi: 10.1128/mbio.02970-21 (PMC9600970; doi:10.1128/mbio.02970-21)
Supplement: TABLE S2 [file mbio.02970-21-s0005.pdf]

Table S2 - Mitochondrial carrier proteins

A list of 35 mitochondrial transporter identified by Palmieri *et al.* (1996) and reviewed by Palmieri and Monné (2016). In this list the systematic name, standard name, importance in this study and the function of the gene encoding for mitochondrial transporters are listed. NA, not assigned. **CRC1* was not considered as involved in the CCM since there is no carnitine supplied to the cultures in this study and thus the carnitine shuttle is not active.

Systematic name	Standard name	Considered as involved in CCM?	Function
**YIL006w**	*NDT1*	YES	Mitochondrial NAD^+ transporter
**YEL006w**	*NDT2*	YES	Mitochondrial NAD^+ transporter
**YIL134w**	*FLX1*	YES	Mitochondrial FAD transporter
**YBR192w**	*RIM2*	NO	Mitochondrial pyrimidine nucleotide transporter
**YDL119c**	*HEM25*	NO	Mitochondrial glycine transporter
**YNL003c**	*SAM5*	NO	S-adenosylmethionine transporter of the mitochondrial inner membrane
**YKR052c**	*MRS4*	NO	Iron transporter of the mitochondrial carrier family (may transport other cations)
**YJL133w**	*MRS3*	NO	Iron transporter of the mitochondrial carrier family (May transport other cations)
**YGR257c**	*MTM1*	NO	pyridoxal 5'-phosphate (PLP) transporter
**YER053c**	*PIC2*	NO	Mitochondrial copper and phosphate carrier
**YJR077c**	*MIR1*	NO	Mitochondrial phosphate carrier
**YOR130c**	*ORT1*	NO	Ornithine transporter of the mitochondrial inner membrane (ornithine-proton exchange or ornithine-ornithine exchange also transports arginine and lysine)
**YOR100c**	*CRC1*	NO*	Mitochondrial inner membrane carnitine transporter. Transports carnitine, acetylcarnitin and propionylcarnitine .
**YBR104w**	*YMC2*	NO	Putative mitochondrial inner membrane transporter. Proposed role in oleate metabolism and glutamate biosynthesis.
**YPR058w**	*YMC1*	NO	Secondary mitochondrial inner membrane glycine transporter; required with HEM25 for the transport of glycine into mitochondria. Proposed role in oleate metabolism and glutamate biosynthesis.
**YPL134c**	*ODC1*	YES	2-oxodicarboxylate transporter (transports 2-oxoglutarate and oxodipate+ corresponding dicarboxylates and malate by counterexchange)
**YOR222w**	*ODC2*	YES	2-oxodicarboxylate transporter (transports oxogluterate and oxodipate+ corresponding dicarboxylates and malate by counterexchange)
**YPR021c**	*AGC1*	NO	Mitochondrial amino acid transporter (transport aspartate and glutamate in uniport as well as in exchange mechanism)
**YJR095w**	*SFC1*	YES	Mitochondrial succinate-fumarate counter exchange transporter (fumerate to cytosol, succinate to mitochondria)
**YBR291c**	*CTP1*	YES	Mitochondrial inner membrane citrate transporter
**YFR045w**	NA	NO	Putative mitochondrial transport protein; null mutant is viable
**YMR241w**	*YHM2*	YES	Citrate and oxoglutarate carrier protein (Citrate exported and oxogluterate imported) (oxaloacetate, succinate and fumerate to a lesser extend)

**YLR348c**	*DIC1*	YES	Mitochondrial dicarboxylate carrier (transports malate, succinate, malonate, inorganic phosphate by counter exchange mechanism. Also sulphate, thiosulphate)
**YKL120w**	*OAC1*	YES	Transports oxaloacetate and sulfate (unidirectional+counterexchange)
**YDL198c**	*GGC1*	NO	Mitochondrial GTP/GDP exchange transporter, essential for mitochondrial genome maintenance, has a role in mitochondrial iron transport
**YGR096w**	*TPC1*	YES	Mediates uptake of the essential cofactor thiamine pyrophosphate (ThPP) into mitochondria
**YMR056c**	*AAC1*	YES	Mitochondrial inner membrane ADP/ATP translocator
**YBL030c**	*AAC2*	YES	Mitochondrial inner membrane ADP/ATP translocator
**YBR085w**	*AAC3*	YES	Mitochondrial inner membrane ADP/ATP translocator
**YHR002w**	*LEU5*	YES	Involved in the accumulation of CoA in the mitochondrial matrix
**YPR011c**	NA	NO	Putative 5'-phosphosulfate (APS) and 3'-phospho-adenosine 5'-phosphosulfate (PAPS) transporter. Not enough information on the function to include in this study.
**YNL083w**	*SAL1*	YES	ADP/ATP transporter (activity of either Sal1p or Pet9p is critical for viability)
**YGL080w**	*MPC1*	YES	Conserved subunit of mitochondrial pyruvate carrier (MPC)
**YHR162w**	*MPC2*	YES	Highly conserved subunit of the mitochondrial pyruvate carrier (MPC)
**YGR243w**	*MPC3*	YES	Highly conserved subunit of the mitochondrial pyruvate carrier (MPC)
**YMR166C**	*MME1*	NO	Mitochondrial inner membrane transporter that exports magnesium

Palmieri F, Monné M. Discoveries, metabolic roles and diseases of mitochondrial carriers: A review. Biochim Biophys Acta 2016;1863: 2362-78.

Palmieri L, Palmieri F, Runswick MJ et al. Identification by bacterial expression and functional reconstitution of the yeast genomic sequence encoding the mitochondrial dicarboxylate carrier protein. FEBS Lett 1996;399: 299-302.
