# Supplementary material for: Top-Down, Knowledge-Based Genetic Reduction of Yeast Central Carbon Metabolism
Source: mBio. 2022 Sep 21;13(5):e02970-21. doi: 10.1128/mbio.02970-21 (PMC9600970; doi:10.1128/mbio.02970-21)

Figure S1 - Growth rate of the *OAC1* deletion mutant

Maximum specific growth rate of S. *cerevisiae* CEN.PK113-7D (naïve reference strain) and the *OAC1* deletion mutant, *S. cerevisiae* IMK588, tested in shake flasks on selective SMD medium. Growth rates represent the average and standard deviation of two biological duplicates. * IMK588 had a 26% slower growth rate with respect to CEN.PK113-7D (two-tailed paired homoscedastic t-test $p<0.05$).

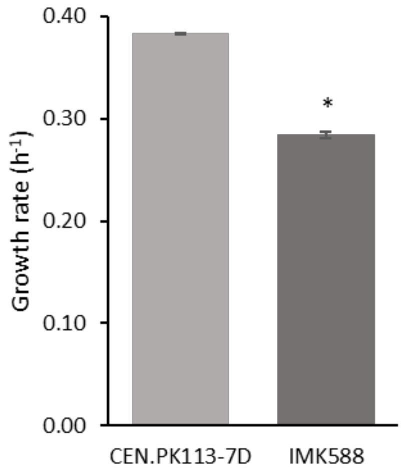

Supplement: FIG S1 [file mbio.02970-21-s0001.pdf]
