# Supplementary material for: Top-Down, Knowledge-Based Genetic Reduction of Yeast Central Carbon Metabolism
Source: mBio. 2022 Sep 21;13(5):e02970-21. doi: 10.1128/mbio.02970-21 (PMC9600970; doi:10.1128/mbio.02970-21)
Supplement: FIG S2 [file mbio.02970-21-s0002.pdf]

Figure S2 - Growth rates of deletion strains

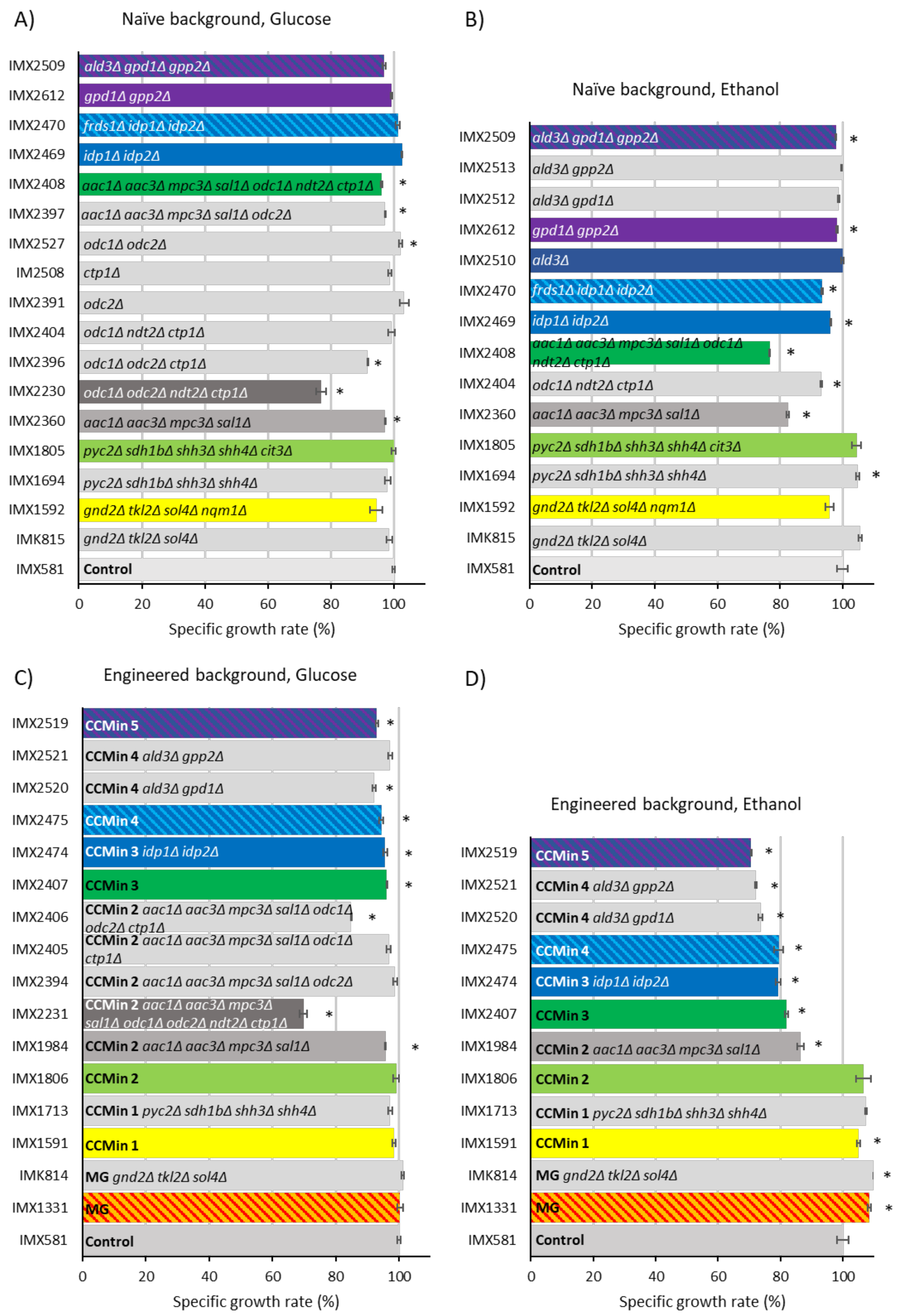

Maximum specific growth rates of all *S. cerevisiae* strains tested in shake flasks on selective SMD (A, B) or SME (B, D) supplemented with uracil. Represented are deletion strains in naïve background (A, B) and in the engineered background (C,D). Colour coding is according to Figure 2 in the main manuscript. Growth rates represent the average and standard deviation of two biological duplicates. Growth rates of the deletion strains are expressed as % of the control strain IMX581. Significant changes in growth rate with respect to the control strain are indicated with a * (two-tailed paired homoscedastic t-test $p<0.05$).
