# Supplementary material for: Top-Down, Knowledge-Based Genetic Reduction of Yeast Central Carbon Metabolism
Source: mBio. 2022 Sep 21;13(5):e02970-21. doi: 10.1128/mbio.02970-21 (PMC9600970; doi:10.1128/mbio.02970-21)

Figure S3 - Growth rates in specific environments

A, B, C and D) Specific growth rate determination on osmotic stress conditions (supplemented with uracil) measured with the growth profiler in biological triplicate. E) Specific growth rate of *FRDS1* and *AAC3* deletion mutants on SMD supplemented with Tween, ergosterol and uracil measured under anaerobic conditions in shake flasks in biological duplicate. F) Specific growth rate of *MPC3* complementation strains on SME supplemented with uracil in shake flask in biological duplicate. G) Specific growth rate of *MPC3* complementation strain on SM with 83.3 mM pyruvate supplemented with uracil in shake flask in biological duplicate. Significant changes in growth rate with respect to the parental strain are indicated with a * (two-tailed paired homoscedastic t-test p<0.05).

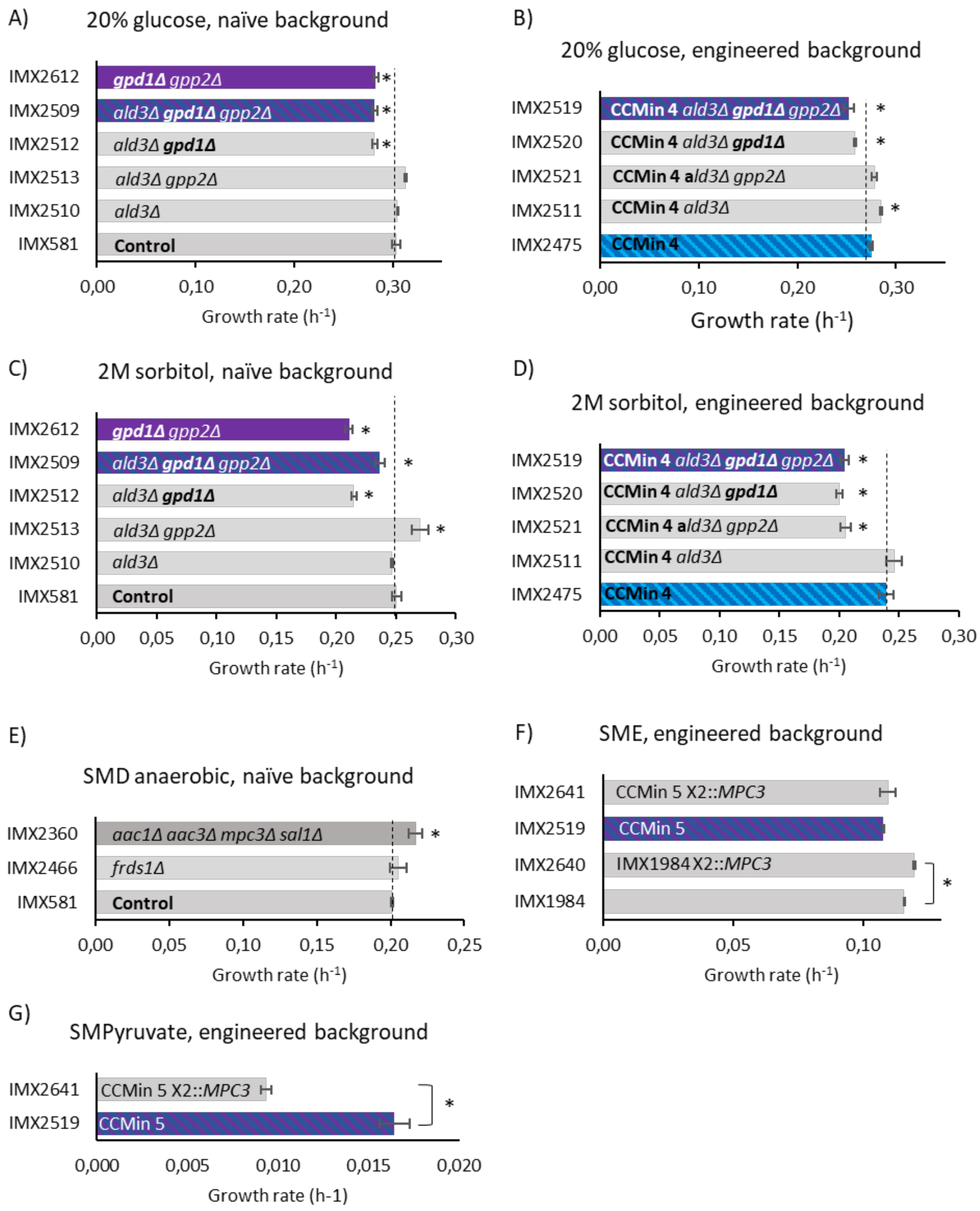

Supplement: FIG S3 [file mbio.02970-21-s0003.pdf]
