# Supplementary material for: Top-Down, Knowledge-Based Genetic Reduction of Yeast Central Carbon Metabolism
Source: mBio. 2022 Sep 21;13(5):e02970-21. doi: 10.1128/mbio.02970-21 (PMC9600970; doi:10.1128/mbio.02970-21)
Supplement: TABLE S3 [file mbio.02970-21-s0006.pdf]

Table S3 - Strains

Name (Accession no.)	Relevant genotype	Reduced pathway	Origin
	**Unreduced background**		
**CEN.PK113-7D**	*MATa ura3-52 can1Δ::cas9-natNT2 TRP1 LEU2 HIS3*		(Entian and Kötter 2007)
**IMX581 (Y40593)**	*MATa ura3-52 can1Δ::cas9-natNT2 TRP1 LEU2 HIS3*		(Mans *et al.* 2015)
**IMK588**	*MATa URA3 HIS3 LEU2 TRP1 OAC1Δ::kanMX*		This study
**IMK815**	*MATa ura3-52 HIS3 LEU2 TRP1 can1Δ::cas9-natNT2 gnd2Δ tkl2Δ sol4Δ*		This study
**IMX1592**	*MATa ura3-52 HIS3 LEU2 TRP1 can1Δ::cas9-natNT2 gnd2Δ tkl2Δ sol4Δ nqm1Δ*	Pentose phosphate pathway	This study
**IMX1694**	*MATa ura3-52 can1Δ::cas9-natNT2 TRP1 LEU2 HIS3 pyc2Δ sdh1bΔ shh3Δ shh4Δ*		This study
**IMX1805**	*MATa ura3-52 can1Δ::cas9-natNT2 TRP1 LEU2 HIS3 pyc2Δ sdh1bΔ shh3Δ shh4Δ cit3Δ*	Anaplerotic reaction and Krebs cycle	This study
**IMX2230**	*MATa ura3-52 HIS3 LEU2 TRP1 MAL2-8c SUC2 can1::CAS9-nat odc1Δ odc2Δ ndt2Δ ctp1Δ*		This study
**IMX2360**	*MATa ura3-52 HIS3 LEU2 TRP1 MAL2-8c SUC2 can1::CAS9-nat aac1Δ aac3Δ sal1Δ mpc3Δ*		This study
**IMX2391**	*MATa ura3-52 HIS3 LEU2 TRP1 MAL2-8c SUC2 can1::CAS9-nat odc2Δ*		This study
**IMX2396**	*MATa ura3-52 HIS3 LEU2 TRP1 MAL2-8c SUC2 can1::CAS9-nat ctp1Δ odc1Δ odc2Δ*		This study
**IMX2397**	*MATa ura3-52 HIS3 LEU2 TRP1 MAL2-8c SUC2 can1::CAS9-nat aac1Δ aac3Δ mpc3Δ sal1Δ odc2Δ*		This study
**IMX2404**	*MATa ura3-52 HIS3 LEU2 TRP1 MAL2-8c SUC2 can1::CAS9-nat ctp1Δ odc1Δ ndt2Δ*		This study
**IMX2408**	*MATa ura3-52 HIS3 LEU2 TRP1 MAL2-8c SUC2 can1::CAS9-nat aac1Δ aac3Δ mpc3Δ sal1Δ ctp1Δ odc1Δ ndt2Δ*	Mitochondrial transporters	This study
**IMX2416**	*MATa ura3-52 HIS3 LEU2 TRP1 MAL2-8c SUC2 can1::CAS9-nat aac1Δ aac3Δ mpc3Δ sal1Δ ctp1Δ odc1Δ odc2Δ*		This study
**IMX2508**	*MATa ura3-52 HIS3 LEU2 TRP1 MAL2-8c SUC2 can1::CAS9-nat ctp1Δ*		This study
**IMX2527**	*MATa ura3-52 HIS3 LEU2 TRP1 MAL2-8c SUC2 can1::CAS9-nat odc1Δ odc2Δ*		This study
**IMX2466**	*MATa ura3-52 HIS3 LEU2 TRP1 MAL2-8c SUC2 can1::CAS9-nat frds1Δ*	Fumarate reductase	This study

**IMX2467**	*MATa ura3-52 HIS3 LEU2 TRP1 MAL2-8c SUC2 can1::CAS9-nat idp1Δ*		This study
**IMX2468**	*MATa ura3-52 HIS3 LEU2 TRP1 MAL2-8c SUC2 can1::CAS9-nat idp2Δ*		This study
**IMX2469**	*MATa ura3-52 HIS3 LEU2 TRP1 MAL2-8c SUC2 can1::CAS9-nat idp1Δ idp2Δ*	Glyoxylate cycle	This study
**IMX2470**	*MATa ura3-52 HIS3 LEU2 TRP1 MAL2-8c SUC2 can1::CAS9-nat frds1Δ idp1Δ idp2Δ*		This study
**IMX2509**	*MATa ura3-52 HIS3 LEU2 TRP1 MAL2-8c SUC2 can1::CAS9-nat ald3Δ gpd1Δ gpp2Δ*		This study
**IMX2510**	*MATa ura3-52 HIS3 LEU2 TRP1 MAL2-8c SUC2 can1::CAS9-nat ald3Δ*	Acetyl-CoA synthesis	This study
**IMX2512**	*MATa ura3-52 HIS3 LEU2 TRP1 MAL2-8c SUC2 can1::CAS9-nat ald3Δ gpd1Δ*		This study
**IMX2513**	*MATa ura3-52 HIS3 LEU2 TRP1 MAL2-8c SUC2 can1::CAS9-nat ald3Δ gpp2Δ*		This study
**IMX2612**	*MATa ura3-52 HIS3 LEU2 TRP1 MAL2-8c SUC2 can1::CAS9-nat gpd1Δ gpp2Δ*	Glycerol synthesis	This study
	**Reduced background**		
**IMX370**	*MATa ura3-52 his3-1 leu2-3,112 MAL2-8c SUC2 glk1::HIS5, hxk1::LEU2, tdh1, tdh2::AB, gpm2::LoxP, gpm3, eno1, pyk2, pdc5, pdc6, adh2, adh5, adh4*	Glycolysis	(Solis-Escalante *et al.* 2015)
**IMX1331 (MG)**	*MATa ura3-52 his3-1 leu2-3,112 MAL2-8c SUC2 glk1::Sphis5 hxk1::KlLEU2 tdh1 tdh2 gpm2 gpm3 eno1 pyk2 pdc5 pdc6 adh2 adh5 adh4 can1Δ::cas9-natNT2*	Glycolysis	This study
**IMK814**	*MATa ura3-52 his3-1 leu2-3,112 MAL2-8c SUC2 glk1::Sphis5 hxk1::KlLEU2 tdh1Δ tdh2Δ gpm2Δ gpm3Δ eno1Δ pyk2Δ pdc5Δ pdc6Δ adh2Δ adh5Δ adh4Δ can1Δ::cas9-natNT2 gnd2Δ tkl2Δ sol4Δ*		This study
**IMX1591 (CCMin 1)**	*MATa ura3-52 his3-1 leu2-3,112 MAL2-8c SUC2 glk1::Sphis5 hxk1::KlLEU2 tdh1Δ tdh2Δ gpm2Δ gpm3Δ eno1Δ pyk2Δ pdc5Δ pdc6Δ adh2Δ adh5Δ adh4Δ can1Δ::cas9-natNT2 gnd2Δ tkl2Δ sol4Δ nqm1Δ*	Pentose phosphate pathway	This study
**IMX1713**	*MATa ura3-52 his3-1 leu2-3,112 MAL2-8c SUC2 glk1::Sphis5 hxk1::KlLEU2 tdh1Δ tdh2Δ gpm2Δ gpm3Δ eno1Δ pyk2Δ pdc5Δ pdc6Δ adh2Δ adh5Δ adh4Δ can1Δ::cas9-natNT2 gnd2Δ tkl2Δ sol4Δ nqm1Δ pyc2Δ sdh1bΔ shh3Δ shh4Δ*		This study
**IMX1806 (CCMin 2)**	*MATa ura3-52 his3-1 leu2-3,112 MAL2-8c SUC2 glk1::Sphis5 hxk1::KlLEU2 tdh1Δ tdh2Δ gpm2Δ gpm3Δ eno1Δ pyk2Δ pdc5Δ pdc6Δ adh2Δ adh5Δ adh4Δ can1Δ::cas9-natNT2 gnd2Δ tkl2Δ sol4Δ nqm1Δ pyc2Δ sdh1bΔ shh3Δ shh4Δ cit3Δ*	Anaplerotic reaction and Krebs cycle	This study

**IMX1984**	*MATa ura3-52 his3-1 leu2-3,112 MAL2-8c SUC2 glk1::Sphis5 hxk1::KlLEU2 tdh1Δ tdh2Δ gpm2Δ gpm3Δ eno1Δ pyk2Δ pdc5Δ pdc6Δ adh2Δ adh5Δ adh4Δ can1Δ::cas9-natNT2 gnd2Δ tkl2Δ sol4Δ nqm1Δ pyc2Δ sdh1bΔ shh3Δ shh4Δ cit3Δ aac1Δ aac3Δ sal1Δ mpc3Δ*		This study
**IMX2231**	*MATa ura3-52 his3-1 leu2-3,112 MAL2-8c SUC2 glk1::Sphis5 hxk1::KlLEU2 tdh1Δ tdh2Δ gpm2Δ gpm3Δ eno1Δ pyk2Δ pdc5Δ pdc6Δ adh2Δ adh5Δ adh4Δ can1Δ::cas9-natNT2 gnd2Δ tkl2Δ sol4Δ nqm1Δ pyc2Δ sdh1bΔ shh3Δ shh4Δ cit3Δ aac1Δ aac3Δ sal1Δ mpc3Δ odc1Δ odc2Δ ndt2Δ ctp1Δ*		This study
**IMX2394**	*MATa ura3-52 his3-1 leu2-3,112 MAL2-8c SUC2 glk1::Sphis5 hxk1::KlLEU2 tdh1Δ tdh2Δ gpm2Δ gpm3Δ eno1Δ pyk2Δ pdc5Δ pdc6Δ adh2Δ adh5Δ adh4Δ can1Δ::cas9-natNT2 gnd2Δ tkl2Δ sol4Δ nqm1Δ pyc2Δ sdh1bΔ shh3Δ shh4Δ cit3Δ aac1Δ aac3Δ sal1Δ mpc3Δ odc2Δ*		This study
**IMX2405**	*MATa ura3-52 his3-1 leu2-3,112 MAL2-8c SUC2 glk1::Sphis5 hxk1::KlLEU2 tdh1Δ tdh2Δ gpm2Δ gpm3Δ eno1Δ pyk2Δ pdc5Δ pdc6Δ adh2Δ adh5Δ adh4Δ can1Δ::cas9-natNT2 gnd2Δ tkl2Δ sol4Δ nqm1Δ pyc2Δ sdh1bΔ shh3Δ shh4Δ cit3Δ aac1Δ aac3Δ sal1Δ mpc3Δ ctp1Δ odc1Δ*		This study
**IMX2406**	*MATa ura3-52 his3-1 leu2-3,112 MAL2-8c SUC2 glk1::Sphis5 hxk1::KlLEU2 tdh1Δ tdh2Δ gpm2Δ gpm3Δ eno1Δ pyk2Δ pdc5Δ pdc6Δ adh2Δ adh5Δ adh4Δ can1Δ::cas9-natNT2 gnd2Δ tkl2Δ sol4Δ nqm1Δ pyc2Δ sdh1bΔ shh3Δ shh4Δ cit3Δ aac1Δ aac3Δ sal1Δ mpc3Δ ctp1Δ odc1Δ odc2Δ*		This study
**IMX2407 (CCMin 3)**	*MATa ura3-52 his3-1 leu2-3,112 MAL2-8c SUC2 glk1Δ::Sphis5 hxk1Δ::KlLEU2 tdh1Δ tdh2Δ gpm2Δ gpm3Δ eno1Δ pyk2Δ pdc5Δ pdc6Δ adh2Δ adh5Δ adh4Δ can1Δ::cas9-natNT2 gnd2Δ tkl2Δ sol4Δ nqm1Δ pyc2Δ sdh1bΔ shh3Δ shh4Δ cit3Δ aac1Δ aac3Δ sal1Δ mpc3Δ ctp1Δ odc1Δ ndt2Δ*	Mitochondrial transporters	This study
**IMX2471**	*MATa ura3-52 his3-1 leu2-3,112 MAL2-8c SUC2 glk1Δ::Sphis5 hxk1Δ::KlLEU2 tdh1Δ tdh2Δ gpm2Δ gpm3Δ eno1Δ pyk2Δ pdc5Δ pdc6Δ adh2Δ adh5Δ adh4Δ can1Δ::cas9-natNT2 gnd2Δ tkl2Δ sol4Δ nqm1Δ pyc2Δ sdh1bΔ shh3Δ shh4Δ cit3Δ aac1Δ aac3Δ sal1Δ mpc3Δ ctp1Δ odc1Δ ndt2Δ frds1Δ*	Fumarate reductase	This study
**IMX2472**	*MATa ura3-52 his3-1 leu2-3,112 MAL2-8c SUC2 glk1Δ::Sphis5 hxk1Δ::KlLEU2 tdh1Δ tdh2Δ gpm2Δ gpm3Δ eno1Δ pyk2Δ pdc5Δ pdc6Δ adh2Δ adh5Δ adh4Δ can1Δ::cas9-natNT2 gnd2Δ*		This study

	tkl2Δ sol4Δ nqm1Δ pyc2Δ sdh1bΔ shh3Δ shh4Δ cit3Δ aac1Δ aac3Δ sal1Δ mpc3Δ ctp1Δ odc1Δ ndt2Δ idp1Δ		
**IMX2473**	*MATa ura3-52 his3-1 leu2-3,112 MAL2-8c SUC2 glk1Δ::Sphis5 hxk1Δ::KlLEU2 tdh1Δ tdh2Δ gpm2Δ gpm3Δ eno1Δ pyk2Δ pdc5Δ pdc6Δ adh2Δ adh5Δ adh4Δ can1Δ::cas9-natNT2 gnd2Δ tkl2Δ sol4Δ nqm1Δ pyc2Δ sdh1bΔ shh3Δ shh4Δ cit3Δ aac1Δ aac3Δ sal1Δ mpc3Δ ctp1Δ odc1Δ ndt2Δ idp2Δ*		This study
**IMX2474**	*MATa ura3-52 his3-1 leu2-3,112 MAL2-8c SUC2 glk1Δ::Sphis5 hxk1Δ::KlLEU2 tdh1Δ tdh2Δ gpm2Δ gpm3Δ eno1Δ pyk2Δ pdc5Δ pdc6Δ adh2Δ adh5Δ adh4Δ can1Δ::cas9-natNT2 gnd2Δ tkl2Δ sol4Δ nqm1Δ pyc2Δ sdh1bΔ shh3Δ shh4Δ cit3Δ aac1Δ aac3Δ sal1Δ mpc3Δ ctp1Δ odc1Δ ndt2Δ idp1Δ idp2Δ*		This study
**IMX2475 (CCMin 4)**	*MATa ura3-52 his3-1 leu2-3,112 MAL2-8c SUC2 glk1Δ::Sphis5 hxk1Δ::KlLEU2 tdh1Δ tdh2Δ gpm2Δ gpm3Δ eno1Δ pyk2Δ pdc5Δ pdc6Δ adh2Δ adh5Δ adh4Δ can1Δ::cas9-natNT2 gnd2Δ tkl2Δ sol4Δ nqm1Δ pyc2Δ sdh1bΔ shh3Δ shh4Δ cit3Δ aac1Δ aac3Δ sal1Δ mpc3Δ ctp1Δ odc1Δ ndt2Δ frds1Δ idp1Δ idp2Δ*	Glyoxylate cycle	This study
**IMX2511**	*MATa ura3-52 his3-1 leu2-3,112 MAL2-8c SUC2 glk1Δ::Sphis5 hxk1Δ::KlLEU2 tdh1Δ tdh2Δ gpm2Δ gpm3Δ eno1Δ pyk2Δ pdc5Δ pdc6Δ adh2Δ adh5Δ adh4Δ can1Δ::cas9-natNT2 gnd2Δ tkl2Δ sol4Δ nqm1Δ pyc2Δ sdh1bΔ shh3Δ shh4Δ cit3Δ aac1Δ aac3Δ sal1Δ mpc3Δ ctp1Δ odc1Δ ndt2Δ frds1Δ idp1Δ idp2Δ ald3Δ*	Acetyl-Coa synthesis	This study
**IMX2519 (CCMin 5)**	*MATa ura3-52 his3-1 leu2-3,112 MAL2-8c SUC2 glk1Δ::Sphis5 hxk1Δ::KlLEU2 tdh1Δ tdh2Δ gpm2Δ gpm3Δ eno1Δ pyk2Δ pdc5Δ pdc6Δ adh2Δ adh5Δ adh4Δ can1Δ::cas9-natNT2 gnd2Δ tkl2Δ sol4Δ nqm1Δ pyc2Δ sdh1bΔ shh3Δ shh4Δ cit3Δ aac1Δ aac3Δ sal1Δ mpc3Δ ctp1Δ odc1Δ ndt2Δ frds1Δ idp1Δ idp2Δ ald3Δ gpd1Δ gpp2Δ*	Glycerol synthesis	This study
**IMX2520**	*MATa ura3-52 his3-1 leu2-3,112 MAL2-8c SUC2 glk1Δ::Sphis5 hxk1Δ::KlLEU2 tdh1Δ tdh2Δ gpm2Δ gpm3Δ eno1Δ pyk2Δ pdc5Δ pdc6Δ adh2Δ adh5Δ adh4Δ can1Δ::cas9-natNT2 gnd2Δ tkl2Δ sol4Δ nqm1Δ pyc2Δ sdh1bΔ shh3Δ shh4Δ cit3Δ aac1Δ aac3Δ sal1Δ mpc3Δ ctp1Δ odc1Δ ndt2Δ frds1Δ idp1Δ idp2Δ ald3Δ gpd1Δ*		This study
**IMX2521**	*MATa ura3-52 his3-1 leu2-3,112 MAL2-8c SUC2 glk1Δ::Sphis5 hxk1Δ::KlLEU2 tdh1Δ tdh2Δ gpm2Δ gpm3Δ eno1Δ pyk2Δ pdc5Δ pdc6Δ*		This study

	adh2Δ adh5Δ adh4Δ can1Δ::cas9-natNT2 gnd2Δ tkl2Δ sol4Δ nqm1Δ pyc2Δ sdh1bΔ shh3Δ shh4Δ cit3Δ aac1Δ aac3Δ sal1Δ mpc3Δ ctp1Δ odc1Δ ndt2Δ frds1Δ idp1Δ idp2Δ ald3Δ gpp2Δ	
**IMX2538 (minimal CCM strain)**	*MATa ura3-52 his3-1 leu2-3,112 MAL2-8c SUC2 glk1Δ::Sphis5 hxk1Δ::KlLEU2 tdh1Δ tdh2Δ gpm2Δ gpm3Δ eno1Δ pyk2Δ pdc5Δ pdc6Δ adh2Δ adh5Δ adh4Δ can1Δ::cas9-natNT2 gnd2Δ tkl2Δ sol4Δ nqm1Δ pyc2Δ sdh1bΔ shh3Δ shh4Δ cit3Δ aac1Δ aac3Δ sal1Δ mpc3Δ ctp1Δ odc1Δ ndt2Δ frds1Δ idp1Δ idp2Δ ald3Δ gpd1Δ gpp2Δ::URA3*	This study
**IMX2640**	*MATa ura3-52 his3-1 leu2-3,112 MAL2-8c SUC2 glk1::Sphis5 hxk1::KlLEU2 tdh1Δ tdh2Δ gpm2Δ gpm3Δ eno1Δ pyk2Δ pdc5Δ pdc6Δ adh2Δ adh5Δ adh4Δ can1Δ::cas9-natNT2 gnd2Δ tkl2Δ sol4Δ nqm1Δ pyc2Δ sdh1bΔ shh3Δ shh4Δ cit3Δ aac1Δ aac3Δ sal1Δ mpc3Δ X2::pMPC3-MPC3-tMPC3*	This study
**IMX2641**	*MATa ura3-52 his3-1 leu2-3,112 MAL2-8c SUC2 glk1Δ::Sphis5 hxk1Δ::KlLEU2 tdh1Δ tdh2Δ gpm2Δ gpm3Δ eno1Δ pyk2Δ pdc5Δ pdc6Δ adh2Δ adh5Δ adh4Δ can1Δ::cas9-natNT2 gnd2Δ tkl2Δ sol4Δ nqm1Δ pyc2Δ sdh1bΔ shh3Δ shh4Δ cit3Δ aac1Δ aac3Δ sal1Δ mpc3Δ ctp1Δ odc1Δ ndt2Δ frds1Δ idp1Δ idp2Δ ald3Δ gpd1Δ gpp2Δ X2::pMPC3-MPC3-tMPC3*	This study

Mans R, van Rossum HM, Wijsman M et al. CRISPR/Cas9: a molecular Swiss army knife for simultaneous introduction of multiple genetic modifications in *Saccharomyces cerevisiae*. FEMS Yeast Res 2015;15: 1-15.

Entian K-D, Kötter P. 25 Yeast Genetic Strain and Plasmid Collections. In: Stansfield I, Stark MJR (eds.) Methods in Microbiology volume 36: Academic Press, 2007, 629-66.

Solis-Escalante D, Kuijpers NG, Barrajon-Simancas N et al. A minimal set of glycolytic genes reveals strong redundancies in *Saccharomyces cerevisiae* central metabolism. Eukaryot Cell 2015;14: 804-16.
