# Supplementary material for: Top-Down, Knowledge-Based Genetic Reduction of Yeast Central Carbon Metabolism
Source: mBio. 2022 Sep 21;13(5):e02970-21. doi: 10.1128/mbio.02970-21 (PMC9600970; doi:10.1128/mbio.02970-21)
Supplement: TABLE S4 [file mbio.02970-21-s0007.pdf]

Table S4 - Plasmids

N.A., not applicable.

Name	Relevant characteristics	Primer(s) used for gRNA	Origin
**p414-*TEF1p-Cas9-CYC1t***	CEN6/ARS ampR *TEF1p-Cas9-CYC1t*	N.A.	(DiCarlo *et al.* 2013)
**pUG-*natNT2***	ampR *AgTEF1p-nat-AgTEF1t*	N.A.	(de Kok *et al.* 2012)
**pUG6**	ampR *AgTEF1p-kanMX-AgTEF1t*	N.A.	(Güldener *et al.* 1996)
**pMEL10**	2µm ampR *KlURA3* gRNA-*CAN1*.Y	N.A.	(Mans *et al.* 2015)
**pMEL11**	2µm ampR *amdSYM* gRNA-*CAN1*.Y	N.A	(Mans *et al.* 2015)
**pROS10**	2µm ampR *URA3* gRNA-*CAN1*.Y gRNA-*ADE2*.Y	N.A	(Mans *et al.* 2015)
**pROS11**	2µm ampR *amdSYM* gRNA-*CAN1*.Y gRNA-*ADE2*.Y	N.A	(Mans *et al.* 2015)
**pUDR286**	2µm ampR *URA3* gRNA-*TKL2* gRNA-*SOL4*	9508 & 9503	This study
**pUDR287**	2µm ampR *amdSYM* gRNA-*GND2*	7231	This study
**pUDR351**	2µm ampR *URA3* gRNA-*CIT3*	12537	This study
**pUDR353**	2µm ampR *URA3* gRNA-*NQM1*	12569	This study
**pUDR354**	2µm ampR *URA3* gRNA-*PYC2* gRNA-*SDH1b*	12514 & 12521	This study
**pUDR355**	2µm ampR *amdSYM* gRNA-*SHH3* gRNA-*SHH4*	9446 & 12529	This study
**pUDR376**	2µm ampR *amdSYM* gRNA-*X2* (2x)	N.A.	(Wronska *et al.* 2020)
**pUDR458**	2µm ampR *URA3* gRNA-*AAC1* gRNA-*AAC3*	13820 & 13826	This study
**pUDR460**	2µm ampR *amdSYM* gRNA-*CTP1* gRNA-*NDT2*	9489 & 13853	This study
**pUDR462**	2µm ampR *amdSYM* gRNA-*SAL1* gRNA-*MPC3*	13830 & 13859	This study
**pUDR606**	2µm ampR *amdSYM* gRNA-*ODC1* gRNA-*ODC2*	13840 & 15580	This study
**pUDR686**	2µm ampR *URA3* gRNA-*ODC1* gRNA-*CTP1*	13840 & 9489	This study
**pUDR687**	2µm ampR *amdSYM* gRNA-*NDT2* (2x)	13853	This study
**pUDR688**	2µm ampR *amdSYM* gRNA-*ODC2* (2x)	13846	This study
**pUDR722**	2µm ampR *amdSYM* gRNA-*FRDS1* (2x)	17280	This study
**pUDR723**	2µm ampR *URA3* gRNA-*IDP1* (2x)	12285	This study
**pUDR724**	2µm ampR *URA3* gRNA-*IDP2* (2x)	17287	This study
**pUDR725**	2µm ampR *URA3* gRNA-*IDP1* gRNA-*IDP2*	12285 & 17287	This study
**pUDR738**	2µm ampR *URA3* gRNA-*CTP1* (2x)	9489	This study
**pUDR739**	2µm ampR *URA3* gRNA-*ALD3* (2x)	17446	This study
**pUDR740**	2µm ampR *amdSYM* gRNA-*GPD1* gRNA-*GPP2*	9498	This study
**pUDR741**	2µm ampR *URA3* gRNA-*GPD1* gRNA-*ALD3*	7772 & 17446	This study
**pUDR742**	2µm ampR *URA3* gRNA-*GPP2* gRNA-*ALD3*	9498 & 17446	This study

DiCarlo JE, Norville JE, Mali P et al. Genome engineering in *Saccharomyces cerevisiae* using CRISPR-Cas systems. Nucleic Acids Res 2013;41: 4336-43.

Mans R, van Rossum HM, Wijsman M et al. CRISPR/Cas9: a molecular Swiss army knife for simultaneous introduction of multiple genetic modifications in *Saccharomyces cerevisiae*. FEMS Yeast Res 2015;15: 1-15.

Wronska AK, Haak MP, Geraats E et al. Exploiting the diversity of *Saccharomycotina* yeasts to engineer biotin-independent growth of *Saccharomyces cerevisiae*. Appl Environ Microbiol 2020;86.

de Kok S, Nijkamp JF, Oud B et al. Laboratory evolution of new lactate transporter genes in a *jen1Δ* mutant of *Saccharomyces cerevisiae* and their identification as *ADY2* alleles by whole-genome resequencing and transcriptome analysis. FEMS Yeast Res 2012;12: 359-74.

Güldener U, Heck S, Fielder T et al. A new efficient gene disruption cassette for repeated use in budding yeast. Nucleic Acids Res 1996;24: 2519-24.
