# Supplementary material for: Top-Down, Knowledge-Based Genetic Reduction of Yeast Central Carbon Metabolism
Source: mBio. 2022 Sep 21;13(5):e02970-21. doi: 10.1128/mbio.02970-21 (PMC9600970; doi:10.1128/mbio.02970-21)
Supplement: TABLE S5 [file mbio.02970-21-s0008.pdf]

Table S5 - List of primers

Primer name	Primer number	Sequence (5' to 3')
**A. pROS/pMEL gRNA primers. gRNA sequence is underlined**		
**2mu inside fw**	5974	TACTTTTGAGCAATGTTTGTGGA
**2mu inside rv**	5975	AACGAGCTACTAAAATATTGCGAA
**Primer_gRNA_426_fw**	5979	TATTGACGCCGGGCAAGAGC
**Primer_stRNA_426_rv**	5980	CGACCGAGTTGCTCTTG
**P426 CRISPR rv**	6005	GATCATTTATCTTTCACTGCGGAGAAG
**RV_gnd2_gRNA**	7231	GTTGATAACGGACTAGCCTTATTTTAACTTGCTATTTCTAGCTCTAAAACT<u>ATGATCTGGCAGCTTCGCGG</u>ATCATTTATCTTTCACTGCGGAGAAGTTTCGAACGCCGAAACATGCGCA
**SOL4_targetRNA FW**	9503	TGCGCATGTTTCGGCGTTCGAAACTTCTCCGCAGTGAAAGATAAATGATC<u>ACATTTTTCCACATATTAAG</u>GTTTTAGAGCTAGAAATAGCAAGTTAAAATAAG
**TKL2_targetRNA FW**	9508	TGCGCATGTTTCGGCGTTCGAAACTTCTCCGCAGTGAAAGATAAATGATC<u>TCAAAAACTTAATGAGGAAT</u>GTTTTAGAGCTAGAAATAGCAAGTTAAAATAAG
**SHH3 gRNA fw**	9446	TGCGCATGTTTCGGCGTTCGAAACTTCTCCGCAGTGAAAGATAAATGATC<u>ATCCGGTTTTTATAACCCCA</u>GTTTTAGAGCTAGAAATAGCAAGTTAAAATAAGGCTAGTCCGTTATCAAC
**CTP1_targetRNA FW**	9489	TGCGCATGTTTCGGCGTTCGAAACTTCTCCGCAGTGAAAGATAAATGATC<u>ACACCAAAATACCATAATAA</u>GTTTTAGAGCTAGAAATAGCAAGTTAAAATAAGGCTAGTCCGTTATCAAC
**GPP2_targetRNA FW**	9498	TGCGCATGTTTCGGCGTTCGAAACTTCTCCGCAGTGAAAGATAAATGATC<u>CAGAACCATATTTGAAAGGC</u>GTTTTAGAGCTAGAAATAGCAAGTTAAAATAAG
**gRNA_IDP1_fwd**	12285	TGCGCATGTTTCGGCGTTCGAAACTTCTCCGCAGTGAAAGATAAATGATC<u>GATCTTATCCCAAATGATAC</u>GTTTTAGAGCTAGAAATAGCAAGTTAAAATAAGGCTAGTCCGTTATCAAC
**PYC2_targetRNA fw**	12514	TGCGCATGTTTCGGCGTTCGAAACTTCTCCGCAGTGAAAGATAAATGATC<u>GTGACTTAAATAAGAAAACT</u>GTTTTAGAGCTAGAAATAGCAAGTTAAAATAAGGCTAGTCCGTTATCAAC
**SDH1b_targetRNA fw**	12521	TGCGCATGTTTCGGCGTTCGAAACTTCTCCGCAGTGAAAGATAAATGATC<u>TTTCTGATAAGTCAATGATC</u>GTTTTAGAGCTAGAAATAGCAAGTTAAAATAAGGCTAGTCCGTTATCAAC
**SHH4_targetRNA fw**	12529	TGCGCATGTTTCGGCGTTCGAAACTTCTCCGCAGTGAAAGATAAATGATC<u>CATATAAGAGGAATCATTTG</u>GTTTTAGAGCTAGAAATAGCAAGTTAAAATAAGGCTAGTCCGTTATCAAC
**CIT3_targetRNA rv**	12537	GTTGATAACGGACTAGCCTTATTTTAACTTGCTATTTCTAGCTCTAAAAC<u>CATTTGTAATGTTCGAATAA</u>GATCATTTATCTTTCACTGCGGAGAAGTTTCGAACGCCGAAACATGCGCA

**NQM1_targetRNA RV**	12569	GTTGATAACGGACTAGCCTTATTTTAACTTGCTATTTCTAGCTCTAAAACTCTAGAACAGTTATATGAATGATCATTTATCTTTCACTGCGGAGAAGTTTCGAACGCCGAAACATGCGCA
**AAC1_targetRNA FW**	13820	TGCGCATGTTTCGGCGTTCGAAACTTCTCCGCAGTGAAAGATAAATGATCGTATAAGAAGACACTGAAAAGTTTTAGAGCTAGAAATAGCAAGTTAAAATAAGGCTAGTCCGTTATCAAC
**AAC3_targetRNA FW**	13826	TGCGCATGTTTCGGCGTTCGAAACTTCTCCGCAGTGAAAGATAAATGATCTTATAAAAAGACCTTGAAATGTTTTAGAGCTAGAAATAGCAAGTTAAAATAAGGCTAGTCCGTTATCAAC
**SAL1_targetRNA FW**	13830	TGCGCATGTTTCGGCGTTCGAAACTTCTCCGCAGTGAAAGATAAATGATCTTGGCATTAACGAAATAAATGTTTTAGAGCTAGAAATAGCAAGTTAAAATAAGGCTAGTCCGTTATCAAC
**ODC1_targetRNA FW**	13840	TGCGCATGTTTCGGCGTTCGAAACTTCTCCGCAGTGAAAGATAAATGATCAAGAATAGTGTTGTGAAAGGGTTTTAGAGCTAGAAATAGCAAGTTAAAATAAGGCTAGTCCGTTATCAAC
**ODC2_targetRNA FW**	13846	TGCGCATGTTTCGGCGTTCGAAACTTCTCCGCAGTGAAAGATAAATGATCGTGCTGTTAAAAAATACAACGTTTTAGAGCTAGAAATAGCAAGTTAAAATAAGGCTAGTCCGTTATCAAC
**ODC2_targetRNA_fw**	15580	TGCGCATGTTTCGGCGTTCGAAACTTCTCCGCAGTGAAAGATAAATGATCTTTTTCAGGGATCTGAAGTAGTTTTAGAGCTAGAAATAGCAAGTTAAAATAAGGCTAGTCCGTTATCAAC
**NDT2_targetRNA FW**	13853	TGCGCATGTTTCGGCGTTCGAAACTTCTCCGCAGTGAAAGATAAATGATCGAAAATTTAAAAATAAGGTTGTTTTAGAGCTAGAAATAGCAAGTTAAAATAAGGCTAGTCCGTTATCAAC
**MPC3_targetRNA FW**	13859	TGCGCATGTTTCGGCGTTCGAAACTTCTCCGCAGTGAAAGATAAATGATCTTATTCACTACATAATAAAAGTTTTAGAGCTAGAAATAGCAAGTTAAAATAAGGCTAGTCCGTTATCAAC
**FRDS1_targetRNA FW**	17280	TGCGCATGTTTCGGCGTTCGAAACTTCTCCGCAGTGAAAGATAAATGATCTAAAGGTGTCCAAGAATTAAGTTTTAGAGCTAGAAATAGCAAGTTAAAATAAGGCTAGTCCGTTATCAAC
**IDP1_targetRNA FW**	12285	TGCGCATGTTTCGGCGTTCGAAACTTCTCCGCAGTGAAAGATAAATGATCGATCTTATCCCAAATGATACGTTTTAGAGCTAGAAATAGCAAGTTAAAATAAGGCTAGTCCGTTATCAAC
**IDP2_targetRNA FW**	17287	TGCGCATGTTTCGGCGTTCGAAACTTCTCCGCAGTGAAAGATAAATGATCATGAGCAAACAAGAATAATCGTTTTAGAGCTAGAAATAGCAAGTTAAAATAAGGCTAGTCCGTTATCAAC
**ALD3_targetRNA FW**	17446	TGCGCATGTTTCGGCGTTCGAAACTTCTCCGCAGTGAAAGATAAATGATCAACTTTAGCTTCTTCTTGATGTTTTAGAGCTAGAAATAGCAAGTTAAAATAAGGCTAGTCCGTTATCAAC
**GPD1_targetRNA FW**	7772	TGCGCATGTTTCGGCGTTCGAAACTTCTCCGCAGTGAAAGATAAATGATCATTCTTCAATCATGTCCGGCGTTTTAGAGCTAGAAATAGCAAGTTAAAATAAG

B. Confirmation of guide RNA plasmids

**FK105-MP1**	2528	TCTTTCCTGCGTTATCCC
**Fus Tag B fw**	4672	CACCTTTCGAGAGGACGATG
**LP crRNA rv**	5941	GCTGGCCTTTTGCTCACATG

**RV_gnd2_gRNA_check**	7257	TATGATCTGGCAGCTTCGCG
**TKL2_beta_dg rv**	9708	ATTCCTCATTAAGTTTTTGA
**SOL4_alpha_dg rv**	9709	CTTAATATGTGGAAAAATGT
**GPP2_beta_dg rv**	9710	GCCTTTCAAATATGGTTCTG
**TEFterm Fw**	9719	TCAAGAACTTGTCATTTGTATAG
**p426_AmdS_origin_removal_2**	10466	GGAAATGTGCGCGGAAC
**fw_CYC1t**	11787	TCATGTAATTAGTTATGTCACGC
**PYC2_pROS_dg rv**	12520	AGTTTTCTTATTTAAGTCAC
**SDH1b_pROS_dg rv**	12527	GATCATTGACTTATCAGAAA
**SHH3_pROS_dg rv**	12528	TGGGGTTATAAAAACCGGAT
**SHH4_pROS_dg rv**	12535	CAAATGATTCCTCTTATATG
**CIT3_pMEL_dg rv**	12542	TTATTCGAACATTACAAATG
**NQM1_pMEL_dg fw**	12729	ATTCATATAACTGTTCTAGA
**AAC1_pROS_dg rv**	13825	TTTTCAGTGTCTTCTTATAC
**AAC3_pROS_dg rv**	13829	ATTTCAAGGTCTTTTTATAA
**SAL1_pROS_dg rv**	13835	ATTTATTTCGTTAATGCCAA
**ODC1_pROS_dg rv**	13845	CCTTTCACAACACTATTCTT
**ODC2_pROS_dg rv**	13851	GTTGTATTTTTTAACAGCAC
**CTP1_pROS_dg rv**	13852	TTATTATGGTATTTTGGTGT
**NDT2_pROS_dg rv**	13858	AACCTTATTTTTAAATTTTC
**MPC3_pMEL_dg fw**	13864	TTATTCACTACATAATAAAA
**FRDS1_pROS_dg rv**	17285	CTTAATTCTTGGACACCTTTAGATC
**IDP1_pROS_dg rv**	17286	CGTATCATTTGGGATAAGATCGATC
**IDP2_pROS_dg rv**	17290	CGATTATTCTTGTTTGCTCATGATC
**GPD1_pROS_dg rv**	17443	TATGGTCGACGTCCTTGCCC
**ALD3_pROS_dg rv**	17447	CATCAAGAAGAAGCTAAAGTTGATC

C. List of primers used for making repair fragments for gene deletion or integration

**OAC1_KO_fw**	6358	ATAGCAAGTCAGACACAAGCACATCTCATCGAATTATATCGTAAGCAAATGCCAGCTGAAGCTTCGTACG
**OAC1_KO_rv**	6359	CTGGCCAATGAATGAAACTTCAAACCTCGGAGTTTGTTATGGGAATTAATGCATAGGCCACTAGTGGATCTG
**Gnd2_repair_FW_new**	7299	AAGAATTCGTAGGTGCAGGTGAGCATATTGCCGGATAAGTGTAGTTACGCAACTACAATTGTTACTAAGGCCCAATCCGGTTGGAGAAGAACTATTGCCCTTGCTGCTACTTACGGTATT
**Gnd2_repair_RV_new**	7300	AATACCGTAAGTAGCAGCAAGGGCAATAGTTCTTCTCCAACCGGATTGGGCCTTAGTAACAATTGTAGTTGCGTAACTACACTTATCCGGCAATATGCTCACCTGCACCTACGAATTCTT
**SHH3 repair oligo fw**	9448	CTCAATGCGACTGTGATAGCTGATAAGTGGAGCTCAGAAATATTCAGAAGCGTAAGAATAATGAAATGTAAGGAATGTACTTAGTGTTTTTTGCTACATAGCTTTTATGATACTCTTTAT
**SHH3 repair oligo rv**	9449	ATAAAGAGTATCATAAAAGCTATGTAGCAAAAAACACTAAGTACATTCCTTACATTTCATTATTCTTACGCTTCTGAATATTTCTGAGCTCCACTTATCAGCTATCACAGTCGCATTGAG
**CTP1_repair oligo fw**	9491	TTGATGTCACAATGAAAGAACTCCAAAGTAGAGCTTGAATTATAAATTAGCATTTTACCGAATGTATTATTGTGTACAATATATCATCTAATGTTTTCTACTCGTTATAAGTCTATTTAC

**CTP1_repair oligo rv**	9492	GTAAATAGACTTATAACGAGTAGAAAACATTAGATGATATAT TGTACACAATAATACATTCGGTAAAATGCTAATTTATAATTCA AGCTCTACTTTGGAGTTCTTTCATTGTGACATCAA
**GPP2_repair oligo fw**	9499	GTTTGCCAAAGGTTTCTTTTCTGCTCAATTTGGTCTAACTCTTT TCATATTAATAGCGCCATTAAATAAATACGTAGATAGATTTTT TTTTTTAAAACATATAGTGTGCTATTATTTCTG
**GPP2_repair oligo rv**	9500	CAGAAATAATAGCACACTATATGTTTTAAAAAAAAAAATCTA TCTACGTATTTATTTAATGGCGCTATTAATATGAAAAGAGTTA GACCAAATTGAGCAGAAAAGAAACCTTTGGCAAAC
**SOL4_repair oligo fw**	9504	CAGCAGTTTTCCAAACAAAGAATGCCATTCATCAAATAATCC ACAACCACCTCAAGAAAATTACACTCGTCTTTATACGAAACT GGCTCCGTTAATCACGACAGACAACCTTAATTACAT
**SOL4_repair oligo rv**	9505	ATGTAATTAAGGTTGTCTGTCGTGATTAACGGAGCCAGTTTC GTATAAAGACGAGTGTAATTTTCTTGAGGTGGTTGTGGATTA TTTGATGAATGGCATTCTTTGTTTGGAAAACTGCTG
**TKL2_repair oligo fw**	9509	TTGTTGGGAGGAGTCCTGAATAAGGAGTGTCGAATATAGGG AGCTTCATTCGTTGTCAAGGAAGTAAACAGTTCTTTGCTATTT CACACTTCCTGGTTGATGGTCACTTGCTGCCTGAAA
**TKL2_repair oligo rv**	9510	TTTCAGGCAGCAAGTGACCATCAACCAGGAAGTGTGAAATA GCAAAGAACTGTTTACTTCCTTGACAACGAATGAAGCTCCCT ATATTCGACACTCCTTATTCAGGACTCCTCCCAACAA
**IDP1_repair_fwd**	12295	TTATGAAATCTTCCTTCAAGCAATTGTGAGACAACAGACGCA CAAGGAAGATCGCCCAGCTCGAATTTACGTAGCCCAATCTAC CACTTTTTTTTTCATTTTTTAAAGTGTTATACTTAG
**IDP1_repair_rev**	12296	CTAAGTATAACACTTTAAAAAATGAAAAAAAAAGTGGTAGA TTGGGCTACGTAAATTCGAGCTGGGCGATCTTCCTTGTGCGT CTGTTGTCTCACAATTGCTTGAAGGAAGATTTCATAA
**PYC2-repair oligo fw**	12516	TGCAAAATAAAGGACAGTTACTAGGAGAGAAAATAAGGGA CATAGAGAACAAAATAAAATTTTTTACTCGTTAATTATATTTT ATGACATCTGAAAATACTAGCTGTACTATATATGGCG
**PYC2-repair oligo rv**	12517	CGCCATATATAGTACAGCTAGTATTTTCAGATGTCATAAAAT ATAATTAACGAGTAAAAAATTTTACTTTGTTCTCTATGTCTCT TATTTTCTCTCCTAGTAACTGTCCTTTATTTTGCA
**SDH1b -repair oligo fw**	12523	GAAAAGAAGGAGCCTAAATACGTATATCTATATACATGTATA CACGTGAGCTAATAAATTTTCTTATTTATTTATTTATTTATTTT GGAGGGCAAACTTATTTATTGATCTGGCAAAAAT
**SDH1b -repair oligo rv**	12524	ATTTTTGCCAGATCAATAAATAAGTTTGCCCTCCAAAATAAAT AAATAAATAAATAAGAAAATTTATTAGCTCACGTGTATACAT GTATATAGATATACGTATTTAGGCTCCTTCTTTTC
**SHH4-repair oligo fw**	12531	AGTTCTAATGAATCAGCAAAGATTCTCAAAAAGGTTGCCCCA ATTCTTAGGAAAGTAGGATCAATATGGTTTGGTTAGTGGTGA CTACCTTTTTTATTCTCGTTATATATGTGTATTAGA
**SHH4-repair oligo rv**	12532	TCTAATACACATATATAACGAGAATAAAAAAGGTAGTCACCA CTAACCAAACCATATTGATCCTACTTTCCTAAGAATTGGGGC AACCTTTTTGAGAATCTTTGCTGATTCATTAGAACT
**CIT3-repair oligo fw**	12538	AAAAAGATCGTATTTGATCAAGAATTTATACATAGACGCCGC TAAATAATTGAATACAAACGCAGTTCCAATTTACAAGAATGC TTCGTTTGCTATTACAATATTGAAATATAAATAAAA

**CIT3-repair oligo rv**	12539	TTTTATTTATATTTCAATATTGTAATAGCAAACGAAGCATTCT TGTAAATTGGAACTGCGTTTGTATTCAATTATTTAGCGGCGT CTATGTATAAATTCTTGATCAAATACGATCTTTTT
**NQM1_repair oligo fw**	12570	TTCTTGCTAGCGTAAGTCATAAAAAATAGGAAATAATCACAT ATATACAAGAAATTAAATTCATTAAGAGTAGAGGTACCTACT TATATATATAAATATATATATACCACTTTCCTTTTC
**NQM1_repair oligo rv**	12571	GAAAAGGAAAGTGGTATATATATATTTATATATATAAGTAGG TACCTCTACTCTTAATGAATTTAATTTCTTGTATATATGTGATT ATTTCCTATTTTTTATGACTTACGCTAGCAAGAA
**AAC1_repair oligo fw**	13821	TTCCTTTTTACAGCAGTAATGTCTCACACAGAAACATAGACTC AGCAGTCACACTTCGGTAAAAAAAAGAAAACAACAAACGAA TAAAATCTAAAAATTCTACATATTCTTGCTATTTAT
**AAC1_repair oligo rv**	13822	ATAAATAGCAAGAATATGTAGAATTTTTAGATTTTATTCGTTT GTTGTTTTCTTTTTTTTACCGAAGTGTGACTGCTGAGTCTATG TTTCTGTGTGAGACATTACTGCTGTAAAAAGGAA
**AAC3_repair oligo fw**	13827	GTATAATTAACTCAATTGAAGACGGTTTACCTGAAGTGATAT ACTGTGCCTTGAGAAACATCAGTTGGATGAAGAAAAAAGTC ATTTTCTCGACTTCTCTTCACCTTTCGATCGATTTGA
**AAC3_repair oligo rv**	13828	TCAAATCGATCGAAAGGTGAAGAGAAGTCGAGAAAATGACT TTTTTCTTCATCCAACTGATGTTTCTCAAGGCACAGTATATCA CTTCAGGTAAACCGTCTTCAATTGAGTTAATTATAC
**SAL1_repair oligo fw**	13831	GGGAAATATTGAATATTTGAAGGGGCAAATAACGTTTAATTC AATCGAATGACGGTCTAATGATAATAACATATGCATATGTAT CAACTAGTGAATTCTATTTAATTATAAACCGCTGCT
**SAL1_repair oligo rv**	13832	AGCAGCGGTTTATAATTAAATAGAATTCACTAGTTGATACAT ATGCATATGTTATTATCATTAGACCGTCATTCGATTGAATTAA ACGTTATTTGCCCCTTCAAATATTCAATATTTCCC
**ODC1_repair oligo fw**	13841	TTTATCTCATTTATTCTCAAGATAGAACTGTAACGAGGGTGA AAGAAAAAGAACTGTAAGTCCTGACGCTCTTTATTTCATTTT GTTGTAGCCCGCCCATACATGTATACGTATATATAT
**ODC1_repair oligo rv**	13842	ATATATATACGTATACATGTATGGGCGGGCTACAACAAAATG AAATAAAGAGCGTCAGGACTTACAGTTCTTTTTCTTTCACCCT CGTTACAGTTCTATCTTGAGAATAAATGAGATAAA
**ODC2_repair oligo fw**	13847	CACTTTTTTACTGGGTTAGATTCTATATAGTCAAGTAATTCGA GGTAGCATCAAAGTAATTATTTATTGTATATAGAATACCTCTT TCCTCTTCAATCTTAACTACGTTATTTCTACGTC
**ODC2_repair oligo rv**	13848	GACGTAGAAATAACGTAGTTAAGATTGAAGAGGAAAGAGG TATTCTATATACAATAAATAATTACTTTGATGCTACCTCGAAT TACTTGACTATATAGAATCTAACCCAGTAAAAAAGTG
**NDT2_repair oligo fw**	13854	GTTGTTCTCAACTTATTTTCATTGTTTGATGAAAAGCGGAATA TACATCACGGCTATATAAGTGAAACAGAAAAATGTACATGAC ACAAGCTGAGGGTCGTAAAAAGCATCTTTCGCAAT
**NDT2_repair oligo rv**	13855	ATTGCGAAAGATGCTTTTTACGACCCTCAGCTTGTGTCATGT ACATTTTTCTGTTTCACTTATATAGCCGTGATGTATATTCCGCT TTTCATCAAACAATGAAAATAAGTTGAGAACAAC
**MPC3_repair oligo fw**	13860	GTCTTTAAGACTATACGCATAAGCATTCAAGACACATAGAAA CACAAACCTATATTTTTATTACGTAAACGATAATATGTTCCTG AACTCGCATTTTTTTAATGATTTTTTATGACCTCT

**MPC3_repair oligo rv**	13861	AGAGGTCATAAAAAATCATTAAAAAAATGCGAGTTCAGGAA CATATTATCGTTTACGTAATAAAAATATAGGTTTGTGTTTCTA TGTGTCTTGAATGCTTATGCGTATAGTCTTAAAGAC
**FRDS1_repair oligo fw**	17281	CATAGCACCCCATTTTTTTTTCTTATCTTCACTTCAACAACCCC TCATCCGTAATTGTAAGGTGGTGCTAGAATCAATGTCAAGGC TCAAGTCATTGGCAAGAACGACGAAAGGCTACTA
**FRDS1_repair oligo rv**	17282	TAGTAGCCTTTCGTCGTTCTTGCCAATGACTTGAGCCTTGACA TTGATTCTAGCACCACCTTACAATTACGGATGAGGGGTTGTT GAAGTGAAGATAAGAAAAAAAAATGGGGTGCTATG
**IDP2_repair oligo fw**	17288	GCTGCTCAGGCACGAGAATAGGAGGTAAGAAGGTAACGTA CGTATATATATAAAATCGTAACTAAAGATTTGGCGCTCATTCT CGGTAAGTCTGAAAGATCCGCTTATGTTACTACCGAG
**IDP2_repair oligo rv**	17289	CTCGGTAGTAACATAAGCGGATCTTTCAGACTTACCGAGAAT GAGCGCCAAATCTTTAGTTACGATTTTATATATATACGTACGT TACCTTCTTACCTCCTATTCTCGTGCCTGAGCAGC
**GPD1_repair oligo fw**	17444	GAAACAATTGTATATTGTACACCCCCCCCCTCCACAAACACA AATATTGATAATATAAAGATTTATTGGAGAAAGATAACATAT CATACTTTCCCCCACTTTTTTCGAGGCTCTTCTATA
**GPD1_repair oligo rv**	17445	TATAGAAGAGCCTCGAAAAAAGTGGGGGAAAGTATGATATG TTATCTTTCTCCAATAAATCTTTATATTATCAATATTTGTGTTT GTGGAGGGGGGGGGTGTACAATATACAATTGTTTC
**ALD3_repair oligo fw**	17450	TGATTTTATTTGTAAATAGTTATCAACGCCGGTGTCGCCTGAT TCTCTACCAATACCACTTTTTCTTTTGGCTAATTTTCTAAATGT GTATAATCTATATCTCTATGATACAAGTCCAAG
**ALD3_repair oligo rv**	17451	CTTGGACTTGTATCATAGAGATATAGATTATACACATTTAGA AAATTAGCCAAAAGAAAAAGTGGTATTGGTAGAGAATCAGG CGACACCGGCGTTGATAACTATTTACAAATAAAATCA
**GPP2_pURA3_fw**	17752	AACCAGCTAGTGTGTACCAGATCAGTGGAAAAACATAAAAC AATAAAAACAATATTCGGATGAGTATTTTCAATAAATTTGTA GAGGACT
**GPP2_tURA3_rv**	17753	CGAATATAGAATAGGACTGTATCTGAGAATTATTACTTAAAT ATGTTCGATTTTAGAGGATTGCTTTTGTTCCACTACTTTTTG
**X2_pMPC3_fw**	18025	TCACAGAGGGATCCCGTTACCCATCTATGCTGAAGATTTATC ATACTATTCCTCCGCTCGTTATTTTGGTCGCCCCTTTAAC
**X2_tMPC3_rv**	18026	GTCATAACTCAATTTGCCTATTTCTTACGGCTTCTCATAAAAC GTCCCACACTATTCAGGATCCTGCTGCTAAAAAGGTC

D. Verification of removal or insertion of a gene

**AAC3 - CTRL FW**	243	GCTTCCAATGGCCTCCTCACCG
**AAC3 - CTRL RV**	244	GGGGGGGAATGCACTTAACAAAGAACC
**URA3_dg rev**	1065	CCCGGTAAAGCATTTCGAAG
**TKL2 dis500 fw**	1360	TCTTAATGGTGGCTCGCTGTC
**TKL2 dis500 rv**	1361	TCAATGCAGCCCATACACTC
**URA3_dg rev Inside**	1741	GAGCCCTTGCATGACAATTC
**GPD1_dg fw**	2016	CCCACCCACACCACCAATAC
**GPD1_dg rev**	2017	CGGACGCCAGATGCTAGAAG
**URA3_dg fw Inside**	2891	CATGGAGGGCACAGTTAAGC

**URA3_dg rev Insert**	4905	TCTTGGAAACGCTGCCCTAC
**OAC1_fw**	6360	AGTCAGGGTCTGCCGATATG
**OAC1_rv**	6361	CTTTCCGGTCCATGTTCGTC
**FW_gnd2KO_check**	7258	TCTGACAGGTGGCAGTTTCC
**RV_gnd2KO_check**	7259	ATCCGAAAGGCGGCAATAGG
**URA3_dg fw**	7372	GGGCAACGGTTCATCATCTCATGG
**FW_x-2_outside**	7376	GGTCTAGGCCTGCATAATCG
**RV_X-2_outside**	7377	TGCGGCATCATGTCTACTTG
**SHH3 dg fw**	9450	GTGACATGGCTCTTGCGTTC
**SHH3 dg rv**	9451	ACTTGCATCGCATCCAGTTC
**CTP1_dg fw**	9493	TGTCTGGGAACTCGGCTAAC
**CTP1_dg rv**	9494	TGGTTGGCTGTTTTTGTGGC
**GPP2_dg fw**	9501	AGTGTTCCGTCCGCGTCATC
**GPP2_dg rv**	9502	GGCGTTACACAGCCCAATCC
**SOL4_dg fw**	9506	GGGTGGACGTTTAAGCATAC
**SOL4_dg rv**	9507	GTATCACCGGGTGAGCTATG
**IDP1_dg_fwd**	12305	GAGAACCCGAGGACGATGTC
**IDP1_dg_rev**	12306	AACAGCCCCCTAAAACACGG
**IDP2_dg_fwd**	12307	GTGGGTTCTCACCGGGTTAG
**IDP2_dg_rev**	12308	ACTACCAGCTGGGATAGGGG
**PYC2_dg fw**	12518	CTGTGATTGGCAGAGAGGGG
**PYC2_dg rv**	12519	AATTCGTTTGCGCCAAGTCC
**SDH1b _dg fw**	12525	CTCCGGACTTATGTGGCTCC
**SDH1b _dg rv**	12526	ACTGAGGGAGAGGGTAAGG
**SHH4_dg fw**	12533	GCGCACTAGTTTGATGCCTG
**SHH4_dg rv**	12534	TGGCCTGCAAACTAAAACGC
**CIT3_dg fw**	12540	AGTGACACACCATGGTAGCG
**CIT3_dg rv**	12541	GACATCGGGGAAGTGGCTAC
**NQM1_dg fw**	12572	CCTTGATCTGGCTCTGGCTC
**NQM1_dg rv**	12573	CGCAAGGTAATTACGCCACG
**AAC1_dg fw**	13823	TTATCGGCGACTCAGCGTAC
**AAC1_dg rv**	13824	TTTGAGAGAGCACCGTCCAC
**SAL1_dg fw**	13833	TACCGGCCTTTCCAATACCC
**SAL1_dg rv**	13834	ATCTGGTTTGCGTTCGCATC
**ODC1_dg fw**	13843	AATGCGGCGACCAGAGATAG
**ODC1_dg rv**	13844	CCCCAGTCAAGTTGCATTGC
**ODC2_dg fw**	13849	GTGCAGTCGCGATCACTTTC
**ODC2_dg rv**	13850	TCCCTGCGATCCGTTATGTG
**NDT2_dg fw**	13856	TTCTCGACATACTCGCGCTG
**NDT2_dg rv**	13857	CAGCTATTTCGGCAGTGCAC
**MPC3_dg fw**	13862	TGGTTTGGTCACGCAAAACC
**MPC3_dg rv**	13863	AAGTAGAAGAAGGCCGCGAC
**FRDS1_dg fw**	17283	TCGCAGGGTTTCTTCACAGG
**FRDS1_dg rv**	17284	ACAATCTTCAGGCCCGTCTG
**ALD3_dg fw**	17448	GCTTAGCTTACATGGCTGCG
**ALD3_dg rv**	17449	TTTAGGGAGGAGACAGGGAG

E. Construction and verification of IMX1331

***Cas9* DNA fragment**		
CAN1DelcassFW	2873	TCAGACTTCTTAACTCCTGTAAAAACAAAAAAAAAAAAAGGCATAGCAATAAGCTGGAGCTCATAGCTTC
A-CYC1t-rv	4653	GTGCCTATTGATGATCTGGCGGAATGTCTGCCGTGCCATAGCCATGCCTTCACATATAGTCCGCAAATTAAAGCCTTCGAG
**natNT2 DNA fragment**		
tagA-pUG	3093	ACTATATGTGAAGGCATGGCTATGGCACGGCAGACATTCCGCCAGATCATCAATAGGCACCTTCGTACGCTGCAGGTCGAC
CAN1 KO rv	5542	CTATGCTACAACATTCCAAAATTTGTCCCAAAAAGTCTTTGGTTCATGATCTTCCCATACGCATAGGCCACTAGTGGATCTG
**Diagnostic primers to check integration**		
**CAN1 cut rv**	5829	AGAAGAGTGGTTGCGAACAGAG
**m-PCR-HR4-RV**	2673	TGAAGTGGTACGGCGATGC
**m-PCR-HR2-FW**	2668	ACGCGTGTACGCATGTAAC
**Reverse primer pUGamds backbone**	8442	CGGGTGACCCGGCGGGGAC
**Nat Ctrl Fw**	2620	GCCGAGCAAATGCCTGCAAATC
**Can1RV**	2615	GAAATGGCGTGGGAATGTGA
