# Supplementary material for: Top-Down, Knowledge-Based Genetic Reduction of Yeast Central Carbon Metabolism
Source: mBio. 2022 Sep 21;13(5):e02970-21. doi: 10.1128/mbio.02970-21 (PMC9600970; doi:10.1128/mbio.02970-21)
Supplement: TABLE S6 [file mbio.02970-21-s0009.pdf]

Table S6 - Strain transformations

Strain	Parental strain	Mutations	gRNA plasmid	Repair fragment
		**Unreduced background**		
**IMK588**	CEN.PK113-7D	*oac1Δ*	-	*KanMX* repair fragment
**IMK815**	IMX581	*gnd2Δ tkl2Δ sol4Δ*	pUDR286 (*TKL2, SOL4*) + pUDR287 (*GND2*)	*SOL4*: 9504 + 9505<br>*TKL2*: 9509 + 9510<br>*GND2*: 7299 + 7300
**IMX1592**	IMK815	*nqm1Δ*	pUDR353 (*NQM1*)	*NQM1*: 12570 + 12571
**IMX1694**	IMX581	*pyc2Δ sdh1bΔ shh3Δ shh4Δ*	pUDR354 (*PYC2, SDH1b*) + pUDR355 (*SHH3, SHH4*)	*PYC2:* 12516 + 12517<br>*SDH1b:* 12523 + 12524<br>*SHH3:* 9448 + 9449<br>*SHH4*: 12531 + 12532
**IMX1805**	IMX1694	*cit3Δ*	pUDR351	*CIT3:* 12538 + 12539
**IMX2230**	IMX581	*odc1Δ odc2Δ ndt2Δ ctp1Δ*	pUDR606 (*ODC1, ODC2*)<br>pUDR460 (*CTP1, NDT2*)	*ODC1*: 13841 + 13842<br>*ODC2*: 13847 + 13848<br>*NDT2*: 13854 + 13855<br>*CTP1*: 9491 + 9492
**IMX2360**	IMX581	*aac1Δ aac3Δ sal1Δ mpc3Δ*	pUDR458 (*AAC1, AAC3*)<br>pUDR462 (*SAL1, MPC3*)	*AAC1*: 13821 + 13822<br>*AAC3*: 13827 + 13828<br>*SAL1*: 13831 + 13832<br>*MPC3*: 13860 + 13861
**IMX2391**	IMX581	*odc2Δ*	pUDR688 (*ODC2*)	*ODC2*: 13847 + 13848
**IMX2396**	IMX581	*ctp1Δ odc1Δ odc2Δ*	pUDR686 (*ODC1, CTP1*)<br>pUDR688 (*ODC2*)	*ODC1*: 13841 + 13842<br>*ODC2*: 13847 + 13848<br>*CTP1*: 9491 + 9492
**IMX2397**	IMX2360	*odc2Δ*	pUDR688 (*ODC2*)	*ODC2*: 13847 + 13848
**IMX2404**	IMX581	*ctp1Δ odc1Δ ndt2Δ*	pUDR686 (*ODC1, CTP1*)<br>pUDR687 (*NDT2*)	*ODC1*: 13841 + 13842<br>*NDT2*: 13854 + 13855<br>*CTP1*: 9491 + 9492
**IMX2408**	IMX2360	*ctp1Δ odc1Δ ndt2Δ*	pUDR686 (*ODC1, CTP1*)<br>pUDR687 (*NDT2*)	*ODC1*: 13841 + 13842<br>*NDT2*: 13854 + 13855<br>*CTP1*: 9491 + 9492
**IMX2416**	IMX2360	*ctp1Δ odc1Δ odc2Δ*	pUDR686 (*ODC1, CTP1*)<br>pUDR688 (*ODC2*)	*ODC1*: 13841 + 13842<br>*ODC2*: 13847 + 13848<br>*CTP1*: 9491 + 9492
**IMX2508**	IMX581	*ctp1Δ*	pUDR738 (*CTP1*)	*CTP1*: 9491 + 9492
**IMX2527**	IMX581	*odc1Δ odc2Δ*	pUDR606 (*ODC1, ODC2*)	*ODC1*: 13841 + 13842<br>*ODC2*: 13847 + 13848
**IMX2466**	IMX581	*frds1Δ*	pUDR722 (*FRDS1*)	*FRDS1*: 17281 + 17282

**IMX2467**	IMX581	*idp1Δ*	pUDR723 (*IDP1*)	*IDP1*: 12295 + 12296
**IMX2468**	IMX581	*idp2Δ*	pUDR724 (*IDP2*)	*IDP2*: 17288 + 17289
**IMX2469**	IMX581	*idp1Δ idp2Δ*	pUDR725 (*IDP1*, *IDP2*)	*IDP1*: 12295 + 12296<br>*IDP2*: 17288 + 17289
**IMX2470**	IMX581	*frds1Δ idp1Δ idp2Δ*	pUDR722 (*FRDS1*)<br>pUDR725 (*IDP1*, *IDP2*)	*FRDS1*: 17281 + 17282<br>*IDP1*: 12295 + 12296<br>*IDP2*: 17288 + 17289
**IMX2509**	IMX581	*ald3Δ gpd1Δ gpp2Δ*	pUDR739 (*ALD3*)<br>pUDR740 (*GPD1, GPP2*)	*ALD3*: 17450 + 17451<br>*GPD1*: 17444 + 17445<br>*GPP2*: 9499 + 9500
**IMX2510**	IMX581	*ald3Δ*	pUDR739 (*ALD3*)	*ALD3*: 17450 + 17451
**IMX2512**	IMX581	*ald3Δ gpd1Δ*	pUDR741 (*ALD3, GPD1*)	*ALD3*: 17450 + 17451<br>*GPD1*: 17444 + 17445
**IMX2513**	IMX581	*ald3Δ gpp2Δ*	pUDR742 (*ALD3, GPP2*)	*ALD3*: 17450 + 17451<br>*GPP2*: 9499 + 9500
**IMX2612**	IMX581	*gpd1Δ gpp2Δ*	pUDR740 (*GPD1, GPP2*)	*GPD1*: 17444 + 17445<br>*GPP2*: 9499 + 9500
		**Reduced background**		
**IMK814**	IMX1331	*gnd2Δ tkl2Δ sol4Δ*	pUDR286 (*TKL2, SOL4*) +<br>pUDR287 (*GND2*)	*SOL4*: 9504 + 9505<br>*TKL2*: 9509 + 9510<br>*GND2*: 7299 + 7300
**IMX1591 (CCMin 1)**	IMK815	*nqm1Δ*	pUDR353 (*NQM1*)	*NQM1*: 12570 + 12571
**IMX1713**	IMX1591	*pyc2Δ sdh1bΔ shh3Δ shh4Δ*	pUDR354 (*PYC2, SDH1b*) +<br>pUDR355 (*SHH3, SHH4*)	*PYC2:* 12516 + 12517<br>*SDH1b:* 12523 + 12524<br>*SHH3:* 9448 + 9449<br>*SHH4*: 12531 + 12532
**IMX1806 (CCMin 2)**	IMX1713	*cit3Δ*	pUDR351	*CIT3:* 12538 + 12539
**IMX1984**	IMX1806	*aac1Δ aac3Δ sal1Δ mpc3Δ*	pUDR458 (*AAC1, AAC3*)<br>pUDR462 (*SAL1, MPC3*)	*AAC1*: 13821 + 13822<br>*AAC3*: 13827 + 13828<br>*SAL1*: 13831 + 13832<br>*MPC3*: 13860 + 13861
**IMX2231**	IMX1984	*odc1Δ odc2Δ ndt2Δ ctp1Δ*	pUDR606 (*ODC1, ODC2*)<br>pUDR460 (*CTP1, NDT2*)	*ODC1*: 13841 + 13842<br>*ODC2*: 13847 + 13848<br>*NDT2*: 13854 + 13855<br>*CTP1*: 9491 + 9492
**IMX2394**	IMX1984	*odc2Δ*	pUDR688 (*ODC2*)	*ODC2*: 13847 + 13848
**IMX2405**	IMX1984	*ctp1Δ odc1Δ*	pUDR686 (*ODC1, CTP1*)	*ODC1*: 13841 + 13842<br>*CTP1*: 9491 + 9492
**IMX2406**	IMX1984	*ctp1Δ odc1Δ odc2Δ*	pUDR686 (*ODC1, CTP1*)<br>pUDR688 (*ODC2*)	*ODC1*: 13841 + 13842<br>*ODC2*: 13847 + 13848<br>*CTP1*: 9491 + 9492
**IMX2407 (CCMin 3)**	IMX1984	*ctp1Δ odc1Δ ndt2Δ*	pUDR686 (*ODC1, CTP1*)<br>pUDR687 (*NDT2*)	*ODC1*: 13841 + 13842<br>*NDT2*: 13854 + 13855<br>*CTP1*: 9491 + 9492

**IMX2471**	IMX2407	*frds1Δ*	pUDR722 (*FRDS1*)	*FRDS1*: 17281 + 17282
**IMX2472**	IMX2407	*idp1Δ*	pUDR723 (*IDP1*)	*IDP1*: 12295 + 12296
**IMX2473**	IMX2407	*idp2Δ*	pUDR724 (*IDP2*)	*IDP2*: 17288 + 17289
**IMX2474**	IMX2407	*idp1Δ idp2Δ*	pUDR725 (*IDP1*, *IDP2*)	*IDP1*: 12295 + 12296<br>*IDP2*: 17288 + 17289
**IMX2475 (CCMin 4)**	IMX2407	*frds1Δ idp1Δ idp2Δ*	pUDR722 (*FRDS1*)<br>pUDR725 (*IDP1*, *IDP2*)	*FRDS1*: 17281 + 17282<br>*IDP1*: 12295 + 12296<br>*IDP2*: 17288 + 17289
**IMX2511**	IMX2475	*ald3Δ*	pUDR739 (*ALD3*)	*ALD3*: 17450 + 17451
**IMX2519 (CCMin 5)**	IMX2475	*ald3Δ gpd1Δ gpp2Δ*	pUDR739 (*ALD3*)<br>pUDR740 (*GPD1, GPP2*)	*ALD3*: 17450 + 17451<br>*GPD1*: 17444 + 17445<br>*GPP2*: 9499 + 9500
**IMX2520**	IMX2475	*ald3Δ gpd1Δ*	pUDR741 (*ALD3, GPD1*)	*ALD3*: 17450 + 17451<br>*GPD1*: 17444 + 17445
**IMX2521**	IMX2475	*ald3Δ gpp2Δ*	pUDR742 (*ALD3, GPP2*)	*ALD3*: 17450 + 17451<br>*GPP2*: 9499 + 9500
**IMX2538 (minimal CCM strain)**	IMX2520	*gpp2Δ::URA3*	-	*URA3* repair fragment
**IMX2640**	IMX1984	*X2::pMPC3-MPC3-tMPC3*	pUDR376 (*X2*)	*MPC3* repair fragment
**IMX2641**	IMX2519	*X2::pMPC3-MPC3-tMPC3*	pUDR376 (*X2*)	*MPC3* repair fragment
